# Indoor Air Quality and Bioaerosols in Spanish University Classrooms

**DOI:** 10.3390/toxics12030227

**Published:** 2024-03-20

**Authors:** Esther Fuentes-Ferragud, Antonio López, Juan Miguel Piera, Vicent Yusà, Salvador Garrigues, Miguel de la Guardia, F. Xavier López Labrador, Marisa Camaró, María Ibáñez, Clara Coscollà

**Affiliations:** 1 Foundation for the Promotion of Health and Biomedical Research in the Valencia Region, FISABIO-Public Health, 21, Avenida Catalunya, 46020 Valencia, Spain; esther.fuentes@fisabio.es (E.F.-F.); al314087@uji.es (J.M.P.); vicent.yusa@fisabio.es (V.Y.); f.xavier.lopez@uv.es (F.X.L.L.); clara.coscolla@fisabio.es (C.C.); 2 Environmental and Public Health Analytical Chemistry, Research Institute for Pesticides and Water, University Jaume I, S/N, Avenida Sos Baynat, 12071 Castelló de la Plana, Spain; ibanezm@uji.es; 3 Public Health Laboratory of Valencia, 21, Avenida Catalunya, 46020 Valencia, Spain; camaro_mar@gva.es; 4 Analytical Chemistry Department, University of Valencia, Edifici Jeroni Muñoz, Dr. Moliner 50, 46100 Burjassot, Spain; salvador.garrigues@uv.es (S.G.); miguel.delaguardia@uv.es (M.d.l.G.); 5 Microbiology Department, University of Valencia Medical School, Av. de Blasco Ibáñez, 13, 46010 Valencia, Spain; 6 CIBERESP, Institute of Health Carlos III, Sinsesio Delgado Street, 4, 28029 Madrid, Spain

**Keywords:** indoor air quality, classrooms, university, respiratory viruses, saliva, non-target screening

## Abstract

A comprehensive study assessed indoor air quality parameters, focusing on relevant air pollutants such as particulate matter (PM_10_ and PM_2.5_), gaseous compounds (CO, CO_2_, formaldehyde, NO_2_) and volatile/semi-volatile organic chemicals, as well as respiratory viruses (including SARS-CoV-2), fungi and bacteria in Spanish university classrooms. Non-target screening strategies evaluated the presence of organic pollutants inside and outside the classrooms. Saliva samples from teachers and students were collected to explore correlations between respiratory viruses in the air and biological samples. Indoor results revealed the punctual exceedance of recommended guidelines for CO_2_, formaldehyde (HCHO), volatile organic compounds (TVOCs) and PM in the least naturally ventilated classrooms. Significant differences occurred between the classes, with the least ventilated one showing higher average concentrations of CO_2_, HCHO, NO_2_, PM_10_ and PM_2.5_. A respiratory virus (rhinovirus/enterovirus) was detected in the medium naturally ventilated classroom, although saliva samples tested negative. Suspect screening tentatively identified 65 substances indoors and over 200 outdoors, with approximately half reporting a high toxicological risk based on the Cramer rules. The study provides a comprehensive overview of indoor air quality, respiratory viruses and organic pollutants in university classrooms, highlighting the variations and potential health risks associated with ventilation differences.

## 1. Introduction

People spend up to 90% of their time indoors, and consequently, indoor air quality (IAQ) inside homes, offices, educational centres, healthcare facilities and other buildings is an important health determinant [1]. In the European Union (EU), indoor air pollution exposure is associated with many non-communicable diseases and an estimated 2 million deaths yearly [2]. Furthermore, inadequate IAQ has a significant impact on human comfort, productivity and effectiveness [1,3].

IAQ in buildings is the result of a complex interplay between building quality and ventilation, outdoor air quality and indoor emissions from cleaning and/or consumer products. As a result, IAQ is affected by pollutants introduced into buildings from the outside, as well as those originating indoors. The main indoor air pollutants are particulate matter (PM), chemical pollutants like nitrogen dioxide (NO_2_), carbon dioxide (CO_2_), ozone (O_3_), total volatile organic compounds (TVOCs) or semi-volatile organic compounds (SVOCs), as well as biological pollutants (mainly viruses, fungi, bacteria, or toxins) [4,5].

In the EU, it is estimated that almost 80% of the disease burden from indoor exposures is caused by exposure to PM_2.5_ [1]. Evidence suggests that indoor PM may be more bioactive than ambient particles due to the presence of endotoxins and other pro-inflammatory components in indoor particles. Air pollution derived from PM causes irritation to the eyes, nose and throat, as well as breathing difficulties [6].

Chemical products can originate from plastic products, cleaning products, electronic and electrical equipment, textiles, furniture, perfumes and personal care products. VOCs, such as formaldehyde (HCHO) and benzene, are emitted from building materials, furniture and paints and may cause significant health effects. Short-term exposure to VOCs may cause skin irritation, dizziness and nausea, whereas longer-term exposure to some VOCs can be carcinogenic [7]. Vehicle exhaust fumes from roads and nearby parking areas is a major contributor to carbon monoxide (CO) and nitrogen oxides in urban areas [8].

Exposure to bioaerosols can cause infectious, toxic or allergic diseases [9]. In fact, fungi can produce more than 300 different mycotoxins, which can have carcinogenic, immunotoxic, cytotoxic and mutagenic effects [10]. Exposure to some Gram-negative and Gram-positive bacteria, endotoxin and actinomycetes, when dispersed through the air, can cause disease by inhalation [11]. Moreover, several viruses, including SARS-CoV-2, can be transmitted through small droplets of saliva that are expelled from the mouth when coughing, sneezing, talking or breathing, as well as through aerosols [12]. The air quality in the areas containing people infected with such viruses can influence the transmission of infection [13]. Several studies have shown that small particles containing viruses can be spread up to 10 m by aerosols in indoor environments [14].

Nowadays, there is no IAQ directive guideline or standard at the EU level, although some European countries like France, Belgium or Germany have drafted national guidelines [4]. Furthermore, there are other laws and technical standards [15,16] that aim to promote the health and well-being of building occupants through increased consideration of air quality and ventilation. On the other hand, chemical legislation also has an impact on IAQ, including the Regulation on the Registration, Authorisation and Restriction of Chemicals [17] and Directive 2011/65/EU, which regulates the restriction of the use of certain hazardous substances in electrical and electronic equipment [18]. Moreover, the World Health Organisation (WHO) has published reports addressing IAQ in relation to dampness and mould [19], pollutants [20], household fuel combustion [21] and setting ambitious goals for air quality to protect public health [22].

According to the literature, only a few studies on IAQ in university environments worldwide have been published [5,23,24,25], and there is a lack of harmonisation in the way IAQ has been assessed. Overall, only CO_2_ has been regularly measured as a proxy indicator of IAQ.

In contrast, the aims of the present work are as follows: (i) to study IAQ in Spanish university classrooms, including the assessment of bioaerosols and SARS-CoV-2 in the air and saliva samples in the pandemic context, and (ii) to study the presence of other pollutants in indoor and outdoor environments.

This study marks the pioneering effort in assessing organic pollutants in university classrooms through non-target screening strategies alongside the examination of bioaerosols and IAQ sensors.

## 2. Site Characterisation and Sampling

### 2.1. Study Population and Site Characterisation

Studies were carried out in three different classrooms belonging to the Chemistry Faculty of the University of Valencia and the School of Technology and Experimental Science of the University Jaume I (Valencian Region, Spain), with different types of natural ventilation, taking into account their number of windows and doors. Sampling was carried out in 2021, during the reopening of Spanish public buildings in the COVID-19 pandemic, and in 2022. There were approximately 20–40 people in the assessed classrooms. The populations under study were students and teachers of the sampled classrooms. The characteristics of the studied classrooms, such as ventilation level, dimensions, classroom volume or the number of doors, are shown in Table 1.

### 2.2. Sampling Air Strategies

A preliminary visit to the sampled facilities was essential in order to select the most suitable sites to be studied. During the first visit, the ventilation of the classrooms, their size, geographical orientation and classroom capacity were checked. Secondly, the researchers informed the universities about the sampling strategy in order to obtain permission to carry out the sampling procedure. Finally, the research team installed the sampling equipment before classes started on the first sampling day. The field strategy used was original, and it is proposed that this applies to indoor air quality studies in educational centres.

#### 2.2.1. Sampling for Monitoring IAQ

The physico-chemical qualities of the indoor air, CO, CO_2_, HCHO, NO_2_, PM and TVOC concentrations were measured with sensors. Additionally, temperature (°C) and relative humidity (RH %) were recorded. All these parameters were monitored using continuous sensors (Aeroqual Series 500) distributed by PCE Iberica S.L. (Tobarra, Albacete, Spain). These sensors undergo annual calibration by the supplying company. The characteristics of the employed sensors are shown in Table S1.

The measurements of these parameters were carried out on three consecutive university lecture days, from approximately 8:30 a.m. to 1:00 p.m., with measurements recorded every 10 min (logging interval) and representing the hours when the students attended classes. The sensors were placed on tripods at a height of approximately 1.5 m (the height corresponding to the student’s breathing zone) and were homogenously distributed around the classroom.

#### 2.2.2. Sampling for Bioaerosols

Two different samplers were employed for sampling respiratory viruses, including SARS-CoV-2. The first device consisted of a Lealand pump operating at a flow rate of 10 L min^−1^ for 2.5 h, collecting a total volume of 1500 L coupled to a three-stage cassette, which contained a 37 mm diameter PTFE (Polytetrafluoroethylene) filter. The second piece of equipment was the MD-8 air scan sampler, which collected aerosols on gelatine filters (3 µm pore size, 80 mm diameter and soluble in water). Two MD-8 air scans were used for virus sampling, with a flow rate of 50 L min^−1^ for 30 min, collecting a total volume of 1500 L. They have been previously employed in indoor hospital environments for sampling MERS-coronavirus (MERS-CoV), SARS-CoV viruses [26] and SARS-CoV-2, providing good yields [27,28].

Microbiological Air Sampler SAS Super IAQ equipment (VWR, Spain) with 90 mm Petri dishes was employed for sampling bacteria and fungi. The multimode working mode was used, with a flow of 100 L min^−1^ for 1 min and a delay of 10 min and 5 intakes, collecting a total air volume of 500 L. Tryptic soy agar (TSA) Petri dishes were used for bacteria growth [9,29] and Sabouraud dextrose agar (SDA) was used for fungal identification [30]. Petri dishes were incubated in an oven at 25 °C for fungi (SDA) and 30 °C for bacteria (TSA) for 3–5 days.

#### 2.2.3. Sampling Surface for Viruses

Six surface samples were collected in each classroom. On each sampling day, two samples were taken: one on the floor and another one on a student’s desk located in the middle of the classroom. A commercial kit was used to analyse the surfaces (Xpress on-Cov-Hygiene site detection kit, VWR Avantor, Barcelona, Spain) [28].

#### 2.2.4. Sampling Indoor Air

Samples were collected using a low-volume active sampler based on an ^®^Aircheck Touch Pump (SKC, Fullerton, CA, USA) coupled to a sorbent tube (XAD-2 OVS^®^) containing a glass fibre filter (GFF), PUF (polyurethane foam) and XAD-2 resin (SKC, Fullerton, CA, USA). An airflow rate of 0.06 m^3^ h^−1^ was applied for 48 h, collecting a total volume of 2.88 m^3^. Three samplers were placed at the front of the classroom near the teacher’s desk. Samples were stored at −20 °C until analysis.

#### 2.2.5. Sampling Outdoor Air

A low-volume active sampler (Digitel DPA-16) at 2.3 m^3^ h^−1^ for 48 h was employed to collect outdoor air samples (the total volume of air collected was approximately 110 m^3^). Samples included a glass fibre filter (to collect the particulate phase) and PUF-XAD2-PUF sorbent (to collect the gaseous phase).

### 2.3. Saliva Samples Collection

Saliva samples were collected from teachers and students in the context of the pandemic situation. In order to perform it properly, participants had to refrain from eating, drinking and smoking for 30 min before sampling. Furthermore, participants needed to disinfect their hands and put on gloves before collecting the biological sample.

A sterile plastic falcon tube with a 50 mL screw cap was used to collect the samples with a zip-lock bag to store the falcon tubes. Sample volumes between 3 and 5 mL were collected, avoiding bubble formation and spillage. The details of participant recruitment, saliva collection and transport to the laboratory are described in Table S2.

Biological sample collection was approved by the Scientific Ethics Committee of FISABIO (reference 1580887), as it was the formalisation of informed consent from each participant.

### 2.4. Statistical Analysis

Spatial comparisons were carried out using one-way ANOVA in order to know if the obtained results in the different classrooms were significantly different (*p* < 0.05 was considered). In positive cases, Tukey’s range test was employed to know which classroom/s showed significant differences. All calculations and statistical analysis were performed using the SPSS 22 package program and Microsoft Excel 2016.

## 3. Sample Treatment and Analysis

### 3.1. Bioaerosols Samples

Sample preparation was performed as described by López et al., 2021 [28]. Briefly, for the cassette filters, this was as follows: 900 µL of RAV1 lysis buffer (Machery-Nagel, Düren, Germany) was added to the cassette and gently shaken for two hours. Next, 750 µL was extracted, and 5 µL of human DNA was added to the samples just before the extraction as an internal control for the quality of the extraction and amplification.

Regarding the gelatine filters (MD-8 air scan sampler), filters were cut by adding 2 mL of lysis buffer RAV1 (Machery-Nagel, Düren, Germany) and 5 µL of human DNA (as internal control) to a 50 mL tube that was heated for 5 min. Then, 200 µL of the sample solution was added to 550 µL of lysis buffer RAV1 and 30 µL of proteinase K (10 mg/mL). Samples were then heated at 56 °C for 10 min.

In both cases (cassette and gelatine filters), total nucleic acids were extracted using the Nucleospin-96 RNA kit (Macherey-Negel, Düren, Germany) on an automated platform (Hamilton Starlet, Hamilton Company, Düren, Germany). The analysis of respiratory viruses in the air was performed as described by López et al., 2021 [28]. For SARS-CoV-2, the detection was performed by RT-PCR, whereas four different screening multiplexes were employed for the other 17 respiratory viruses. Disposable, sterile and RNase/DNase-free materials and equipment were used for quality control. The use of positive, negative and internal controls as a protocol for processing quality control or quality assurance protocols in each analytical batch ensured that the studied viruses were correctly analysed [28].

### 3.2. Saliva Samples

Sample preparation for saliva samples was performed in a BSL2+ laboratory by preparing extraction pools of five samples as follows: 200 µL of five saliva samples were mixed in the pool and it was added to a tube containing 2 mL of Nuclisens lysis buffer (BioMérieux, Lyon, France) and 30 µL of 10 mg/mL proteinase K. The mixtures were incubated at room temperature for at least 30 min to inactivate SARS-CoV-2. Thereafter, total nucleic acids were extracted using an automated silica-based method in an automated platform (Nuclisens e-Mag, BioMérieux, Lyon, France) and eluted in 50 µL. The analysis of respiratory viruses in saliva was performed in the same way as bioaerosol samples.

### 3.3. SARS-CoV-2 in Surfaces

The analysis of SARS-CoV-2 on surfaces was performed in situ using a commercial kit (Xpress on-Cov-Hygiene site detection kit). This specific kit detects possible specific proteins of the virus. In just fifteen minutes, it was possible to qualitatively know whether the assessed surface was positive or negative for SARS-CoV-2 [28].

### 3.4. Fungi and Bacteria

The analysis of fungi and bacteria was carried out using the total number of colonies observed in the Petri dishes. The concentration, expressed as Colony-Forming Units, CFUs per cubic meter, m^3^ was calculated by dividing the number of colonies for bacteria and fungi by the volume of air sampled. The gender identification of fungi was carried out by microscopic visualisation.

### 3.5. Sample Preparation and Analysis for Indoor Samples

The extraction procedure of indoor air samples was based on pressurised fluid extraction (PLE) using an ASE-350 system (Dionex, Sunnyvale, CA, USA). The solvent extraction mixture was dichloromethane/acetone (1:1) under the following conditions: oven temperature = 50 °C, heat = 5 min, static time = 5 min, pressure = 1500 psi, 3 cycles and flush = 75%. Afterward, samples were filtered and concentrated in a Turbo Vap 500 (Zymark, Idstein, Germany) to less than 10 mL and transferred to 10 mL volumetric flasks in order to adjust them to this final volume. Next, two 5 mL aliquots were taken from each sample to obtain two different extracts (one for liquid chromatography (LC) and one for gas chromatography (GC)). Finally, both extracts of each sample were evaporated until dry in a Turbo Vap 10 (Zymark, Idstein, Germany) and redissolved in 1 mL of water/methanol (70:30) for LC and in 0.5 mL of nonane for GC, both coupled to high-resolution mass spectrometry (HRMS).

For the LC-HRMS analysis, liquid chromatography was coupled to an Orbitrap ID-X Tribrid mass spectrometer (Thermo Fisher, Bremen, Germany), using a Hypersil Gold aQ column (100 mm × 2.1 mm, 1.9 µm). A mass spectrometer was employed in the positive and negative modes (ESI + and ESI −). A full scan was acquired at 120,000 FWHM, MS^2^ data at 15,000 FWHM and MS^3^ data were detected at the Ion trap (FHWM < 0.3). For GC-HRMS, a TraceGOLD TG-5MS column (30 m, 0.25 mm, 0.25 µm) was used, and the acquisition was performed in full scan mode with a resolving power of 60,000 FWHM and a mass range from 40 to 500 *m*/*z*. More information about the gradient and temperature programmes are described by López et al., 2022, and Miralles et al., 2021, respectively [31,32].

### 3.6. Sample Preparation and Analysis for Outdoor Samples

For the two types of samples collected (particulate phase and gaseous phase), microwave-assisted extraction (MAE) using ethyl acetate as the extracted solvent was employed, followed by an evaporation and reconstitution step, as described by Fuentes et al., 2021 [33]. Outdoor air samples were analysed using a non-target approach to identify the maximum number of organic pollutants. The analyses were performed in the same way as indoor samples, as described in Section 3.5, “Sample preparation for indoor samples”. In the analysis, quality assurance protocols were included, including process blanks, spiked blank samples, the blank sample and reagent blank.

## 4. Results and Discussion

The analytical results obtained in university classrooms are presented in this section. More specifically, the results are related to the following: (1) IAQ parameters; (2) SARS-CoV-2 and other respiratory viruses in the environment (air and surface), biological samples (saliva), fungi and bacteria in bioaerosol samples; and (3) non-target results from outdoor and indoor air samples.

### 4.1. IAQ Parameters

Overall, IAQ parameters provided average values below those recommended by the guidelines, including comfort parameters such as temperature and relative humidity (see Table 2). Nevertheless, in classroom 2 (the worst naturally ventilated one), the running concentrations of some pollutants (CO_2_, HCHO, PM_10_, PM_2.5_ and TVOCs) sometimes exceeded the maximum recommended values (MRV) [22,34,35,36]. This fact was probably related to the reduced natural ventilation, the large number of people in relation to the classroom volume, the reduced number of doors and windows or its location in front of a street. In classroom 1 (medium natural ventilation classroom), two of the assessed pollutants (CO_2_ and formaldehyde) showed a peak value higher than the MRV on one of the assessed days. On the other hand, the classroom with the highest natural ventilation (classroom 3) did not show any value higher than the recommended guidelines (see Figures S1–S26). It is also noteworthy that a slight increase was observed in all particle concentrations from day one to day three, except in the classroom with high natural ventilation.

**Figure 1:**
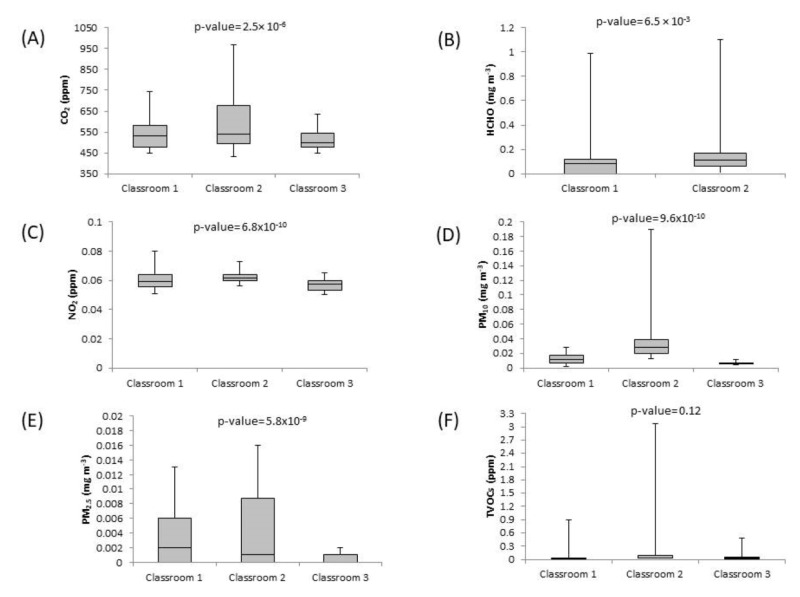
Boxplots for the assessed pollutants in each classroom: (**A**) CO_2_ (ppm), (**B**) HCHO (mg m^−3^), (**C**) NO_2_ (ppm), (**D**) PM_10_ (mg m^−3^), (**E**) PM_2.5_ (mg m^−3^) and (**F**) TVOCs (ppm). Boxplots show the median, minimum and maximum values.

Differences between the studied pollutants in the classrooms were carried out. Significant differences between classrooms were observed for CO_2_, HCHO, NO_2_, PM_10_ and PM_2.5_. For TVOCs and CO only (no levels were observed in two out of three classrooms), no significant differences were found. Looking more closely at the results, it was found that CO_2_ concentrations were higher in the lowest-ventilated classroom (classroom 2) than in classrooms 1 and 3, as an important proxy of poorer air quality. These results were probably related to the large number of people in relation to the volume of the classroom. Particulate matter (PM_10_ and PM_2.5_) levels showed significant differences between the three classrooms, with the highest levels observed in the classroom with the least naturally ventilated room. Concerning NO_2_, concentrations in the best natural ventilated classroom (classroom 3) were significantly lower than in classrooms 1 and 2. Figure 1 shows the obtained box plots for each pollutant in each classroom.

The CO_2_ concentrations determined in our study were similar to those observed in earlier studies at Spanish universities [24]. The average concentration obtained for PM_10_ in classroom 2 was more than two times higher than the average concentrations reported in the study carried out in 2021 [24] and more than ten times higher than the values obtained by Aldekheel et al., 2022, in the USA [23]. Concerning NO_2_, average concentrations were approximately five times higher than the maximum levels obtained by Rodriguez et al., 2022 [24] in Spain. Maximum concentrations measured for TVOCs in classroom 2 were two times higher than the maximum concentration detected in university classrooms in South Africa and in university libraries in Turkey [25,37].

### 4.2. Obtained Results for Bioaerosols in Air, Saliva and Surfaces

SARS-CoV-2 coronavirus was not detected either in the environmental matrices (air and surface) or in the biological samples (saliva). However, one respiratory virus, Human Rhinovirus/Enterovirus (HRV/ENV), was detected one day in the indoor air of classroom 1. Several factors must be taken into account to interpret these results properly. When reopening Spanish public buildings during the COVID-19 pandemic in March 2021, some preventive measures were taken into account by the University, such as no more than 40 people in classrooms with social distancing between them (1.5 m), good ventilation with open doors and windows and mandatory use of masks. Moreover, the incidence of infectious diseases in the Valencian Region was not too high at that time (100–200 new cases per day) [38]. This set of measures could explain the negative results obtained in this study for SARS-CoV-2 in environmental and biological samples. In contrast to our results, a study carried out at the University of Barcelona during the same period (the third wave of the COVID-19 Pandemic in Spain), in which nasal swabs were collected, showed that 1.6% of the students were positive for SARS-CoV-2 [39].

Regarding other respiratory viruses, one positive sample for the HRV/ENV virus was detected in the indoor air on a single day in classroom 1 (see Table S3). By contrast, in the study carried out by Lednicky et al., 2020 [40] in university classrooms in the USA, the HRV/ENV virus was not detected.

For fungal and bacteria bioaerosols, acceptable indoor concentrations were observed. Table 3 shows the results obtained for the fungi and bacteria in the classrooms. The results were lower than the recommended maximum limit of 500 CFU m^−3^ [41] for both bacteria and fungi.

**Figure 2:**
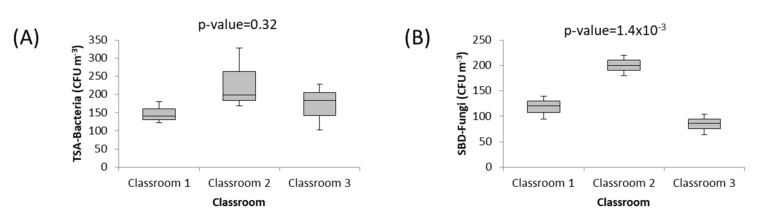
Boxplots for (**A**) TSA bacteria (CFU m^−3^), (**B**) SBD fungi (CFU m^−3^) in the assessed classrooms.

Differences in fungi and bacteria levels obtained in the three classrooms were investigated. Concerning bacteria results, no significant differences were observed between classrooms. On the other hand, fungi results showed that levels in classroom 2 were significantly higher than the obtained results in classrooms 1 and 3 (better-ventilated classrooms). Figure 2 shows the obtained box plots for bacteria (TSA) and fungi (SBD) in the evaluated classrooms.

Bacteria concentrations obtained in the present study were higher than the detected concentrations in a university campus in Turkey during the COVID-19 lockdown [5]. On the other hand, fungi concentrations were in line with those detected in Turkey [5].

Genera identification for fungi was also carried out. Several genera of fungi were identified as Penicillium, Aspergillus, Alternaria, Cladosporium and Mucor or Rhizopus. The obtained results were in line with the fungi genera detected in university classrooms in Italy by Di Giulio et al., 2010 [42], which are quite common in indoor environments and mainly originate in outdoor environments.

### 4.3. Non-Target Air Sample Analysis Results

#### 4.3.1. Indoor

Using a high confidence identification level, 65 substances were tentatively identified in the assessed university classrooms. These 65 substances were not detected in blank samples or their area or were ten or more times higher than the blank samples. According to their toxicological hazard assigned through their Cramer class, 24 were classified as high risk, 4 were classified as medium risk, and 36 were classified as low risk. The obtained results are shown in Table S4. A total of 37 substances were related to industrial activities. It is also noteworthy that some terpenes usually detected in indoor air, like beta-pinene, borneol, camphor or limonene, were detected in the present study. These results are consistent with those obtained by Villanueva et al., 2018 [43] in school areas in the central–southeastern region of Spain.

Furthermore, it is also noticeable that one pesticide (DEET) and one of its metabolites (Cyclopent-2-ene-1-carboxylic acid, 2,3-dimethyl-1-ethyl-, ethyl ester) were tentatively identified in indoor classrooms. This pesticide is currently approved by the EU as a biocide for indoor use.

#### 4.3.2. Outdoor

After analysis of the outdoor samples, 237 compounds were tentatively identified, providing candidates with a high confidence identification level. These 237 substances were not detected in blank samples or their area or were ten or more times higher than the blank samples. According to the toxicological hazard assigned by their Cramer class, 94 substances were classified as low risk, 119 substances were classified as high risk, and 7 substances were classified as intermediate risk. The obtained results are shown in Table S5.

The identified substances were classified, taking into account their sources, common uses or applications. Most of the detected compounds (84 compounds) were related to industrial activities. A major industrial area is located less than 10 km away from one of the sampling points. Other important activities related to the identified substances were pharmaceutical activities (18) and cosmetics (10).

Among the detected substances, it is noteworthy that some authorised pesticides, like pyrimethanil, and other banned pesticides, such as bioresmethrin or carbaryl, were tentatively identified.

Overall, only two substances were tentatively identified in indoor and outdoor environments: 2-methylbenzoic acid and vanillin. Both substances originate from industrial sources.

## 5. Study Limitations

The assessment of classrooms was restricted, and it was only possible to study three classrooms due to the circumstances of the pandemic situation following the reopening of university classrooms. The indoor-to-outdoor ratio was not calculated, as only two substances were identified in both indoor and outdoor environments.

## 6. Conclusions

Efforts to improve IAQ require an integrated approach, with the development and implementation of IAQ monitoring strategies in state and private centres like universities being crucial. In this study, a new strategy to study indoor and outdoor air quality, including bioaerosols in educational centres, is proposed.

Overall, the results showed that the average values provided were below the recommended guidelines for all the pollutants assessed. Nevertheless, some differences were observed between different levels of ventilation in the classrooms, with significant differences (*p* < 0.05) for CO_2_, HCHO, NO_2_, PM_10_, PM_2.5_ and fungi levels in the least naturally ventilated classrooms compared to the better-ventilated classrooms.

Only one respiratory virus (rhinovirus/enterovirus) was detected in the indoor air of the assessed classrooms. Furthermore, during the third wave of the COVID-19 pandemic, students’ and teachers’ saliva was also tested, and no positive results were obtained.

Several substances were tentatively identified in the outdoor and indoor air of the studied classrooms. Some substances that are usually found in indoor environments, like terpenes or in outdoor environments, such as pesticides, were detected in the present study. Overall, about half of the assessed substances in indoor and outdoor environments were classified as high risk, according to the Cramer rules.

Consolidated data at the European level should be made available following policy frameworks that explicitly address indoor air pollution, following harmonized monitoring requirements for sampling, analysis and data interpretation for IAQ in the EU.

## Figures and Tables

**Table 1:** Classroom characteristics.

Assessed Area	Maximum Number of Students	Volume (m^3^)	Natural Ventilation	Number of Doors	Orientation
Classroom 1	32	296.00	Medium	2	East
Classroom 2	37	261.89	Low	1	North
Classroom 3	25	349.67	High	2	West

**Table 2:** IAQ parameter results.

Analytes	Classroom 1				Classroom 2				Classroom 3				
	min	max	Av	SD	min	max	Av	SD	min	max	Av	SD	MRV
**CO (ppm)**	0	0.14	0	0.02	0	1.82	0.09	0.30	0	0	-	-	3.43 ^a^
**CO_2_ (ppm)**	448	**742**	536	67	430	**970**	586	126	448	643	514	44	700 ^b^
**HCHO (mg m^−3^)**	0	**0.99**	0.08	0.08	0	**1.10**	0.16	0.19	- ^1^	- ^1^	- ^1^	- ^1^	0.37 ^c^
**NO_2_ (ppm)**	0.051	0.080	0.061	0.007	0.056	0.073	0.062	0.003	0.050	0.065	0.057	0.004	0.5 ^c^
**PM_10_ (mg m^−3^)**	0.002	0.028	0.013	0.007	0.013	**0.190**	0.033	0.025	0.004	0.011	0.006	0.002	0.045 ^a^
**PM_2.5_ (mg m^−3^)**	0.001	0.015	0.005	0.004	0.003	**0.022**	0.010	0.005	0.002	0.005	0.004	0.001	0.015 ^a^
**TVOCs (ppm)**	0	0.89	0.05	0.12	0.02	**3.07**	0.12	0.36	0	0.50	0.07	0.10	1.32 ^d^
**RH (%)**	32.0	52.1	38.3	8.0	38.9	55.8	47.0	4.1	52.4	66.4	58.8	4.5	30–70 ^c^
**Temperature (°C)**	17.9	22.4	20.6	1.2	18.1	22.0	20.3	1.0	17.8	21.0	19.7	0.8	17–27 ^c^

min = minimum, max= maximum, Av = average, SD = standard deviation, MRV = maximum recommended value, bold: result above the recommended value; ^1^ = results not available due to HCHO sensor problems; ^a^ = WHO guidelines, 2021; ^b^ = LIFTEC and CSIC, 2020; ^c^ = INSHT, 2022; ^d^ = German Supreme Health Authorities, 2012.

**Table 3:** Results of bacteria and fungi in the assessed university classrooms.

	TSA Petri Dishes (Bacteria) (^1^)								
Classroom	Classroom 1			Classroom 2			Classroom 3		
**Sampling Day**	**Day 1**	**Day 2**	**Day 3**	**Day 1**	**Day 2**	**Day 3**	**Day 1**	**Day 2**	**Day 3**
**Colony number**	90	70	61	84	99	164	51	114	92
**CFU m^−3^**	180	140	122	168	198	328	102	228	184
	SBD Petri Dishes (Fungi) (^2^)								
**Classroom**	**Classroom 1**			**Classroom 2**			**Classroom 3**		
**Sampling Day**	**Day 1**	**Day 2**	**Day 3**	**Day 1**	**Day 2**	**Day 3**	**Day 1**	**Day 2**	**Day 3**
**Colony number**	60	47	70	90	110	100	32	43	52
**CFU m^−3^**	120	94	140	180	220	200	64	86	104

(^1^) Incubated at 30 °C; (^2^) incubated at 25 °C.

## Data Availability

Data are contained within article.

## Supplementary Materials

The following supporting information can be downloaded at: https://www.mdpi.com/article/10.3390/toxics12030227/s1, Table S1: Sensors specifications Table S2: Field work manual; Table S3: Results of respiratory viruses in indoor air; Table S4: List of the tentatively identified substances in indoor air; Table S5: List of the tentatively identified substances in outdoor air; Figure S1: CO concentration (ppm) in classroom 1; Figure S2: CO concentration (ppm) in classroom 2; Figure S3: CO concentration (ppm) in classroom 3; Figure S4: CO_2_ concentration (ppm) in classroom 1; Figure S5: CO_2_ concentration (ppm) in classroom 2; Figure S6: CO_2_ concentration (ppm) in classroom 3; Figure S7: HCHO concentration (mg m^−3^) in classroom 1; Figure S8: HCHO concentration (mg m^−3^) in classroom 2; Figure S9: NO_2_ concentration (ppm) in classroom 1; Figure S10: NO_2_ concentration (ppm) in classroom 2; Figure S11: NO_2_ concentration (ppm) in classroom 3; Figure S12: PM_10_ concentration (mg m^−3^) in classroom 1; Figure S13: PM_10_ concentration (mg m^−3^) in classroom 2; Figure S14: PM_10_ concentration (mg m^−3^) in classroom 3; Figure S15: PM_2.5_ concentration (mg m^−3^) in classroom 1; Figure S16: PM_2.5_ concentration (mg m^−3^) in classroom 2; Figure S17: PM_2.5_ concentration (mg m^−3^) in classroom 3; Figure S18: TVOCs concentration (mg m^−3^) in classroom 1; Figure S19: TVOCs concentration (mg m^−3^) in classroom 2; Figure S20: TVOCs concentration (mg m^−3^) in classroom 3; Figure S21: Temperature (°C) in classroom 1; Figure S22: Temperature (°C) in classroom 2; Figure S23: Temperature (°C) in classroom 3; Figure S24: Relative Humidity (%) in classroom 1; Figure S25: Relative Humidity (%) in classroom 2; Figure S26: Relative Humidity (%) in classroom 3.
