# Supplementary material for: Indoor Air Quality and Bioaerosols in Spanish University Classrooms
Source: Toxics. 2024 Mar 20;12(3):227. doi: 10.3390/toxics12030227 (PMC10975315; doi:10.3390/toxics12030227)
Supplement: Supplementary file 1 [file toxics-12-00227-s001.zip › toxics-2873404-supplementary.pdf]

toxics

MDPI

Article

Indoor Air Quality and Bioaerosols in Spanish University Classrooms

Esther Fuentes-Ferragud [1,2], Antonio López [1,*], Juan Miguel Piera [1], Vicent Yusà [1,3,4], Salvador Garrigues [4], Miguel de la Guardia [4], F. Xavier López Labrador [1,5,6], Marisa Camaró [3], María Ibáñez [2] and Clara Coscollà [1]

[1] Foundation for the Promotion of Health and Biomedical Research in the Valencia Region, FISABIO-Public Health, 21, Avenida Catalunya, 46020 Valencia, Spain; esther.fuentes@fisabio.es (E.F.-F.); al314087@uji.es (J.M.P.); vicent.yusa@fisabio.es (V.Y.); f.xavier.lopez@uv.es (F.X.L.L.); clara.coscolla@fisabio.es (C.C.)

[2] Environmental and Public Health Analytical Chemistry, Research Institute for Pesticides and Water, University Jaume I, S/N, Avenida Sos Baynat, 12071 Castelló de la Plana, Spain; ibanezm@uji.es

[3] Public Health Laboratory of Valencia, 21, Avenida Catalunya, 46020 Valencia, Spain; camaro_mar@gva.es

[4] Analytical Chemistry Department, University of Valencia, Edifici Jeroni Muñoz, Dr. Moliner 50, 46100 Burjassot, Spain; salvador.garrigues@uv.es (S.G.); miguel.delaguardia@uv.es (M.d.l.G.)

[5] Microbiology Department, University of Valencia Medical School, Av. de Blasco Ibáñez, 13, 46010 Valencia, Spain

[6] CIBERESP, Institute of Health Carlos III, Sinsesio Delgado Street, 4, 28029 Madrid, Spain

* Correspondence: antonio.lopez@fisabio.es; Tel.: +34-961-925-900

Table of Contents

Type	Captions	Page
Table S1	Sensor specifications	3
Table S2	Field work manual	4
Table S3	Results of respiratory viruses in indoor air	6
Table S4	List of the tentatively identified substances in indoor air	7
Table S5	List of the tentatively identified substances in outdoor air	10
Figure S1	CO concentration (ppm) in classroom 1	19
Figure S2	CO concentration (ppm) in classroom 2	20
Figure S3	CO concentration (ppm) in classroom 3	21
Figure S4	CO_2 concentration (ppm) in classroom 1	22
Figure S5	CO_2 concentration (ppm) in classroom 2	23
Figure S6	CO_2 concentration (ppm) in classroom 3	24
Figure S7	HCHO concentration (mg m^{-3}) in classroom 1	25
Figure S8	HCHO concentration (mg m^{-3}) in classroom 2	26
Figure S9	NO_2 concentration (ppm) in classroom 1	27
Figure S10	NO_2 concentration (ppm) in classroom 2	28
Figure S11	NO_2 concentration (ppm) in classroom 3	29
Figure S12	PM_{10} concentration (mg m^{-3}) in classroom 1	30
Figure S13	PM_{10} concentration (mg m^{-3}) in classroom 2	31
Figure S14	PM_{10} concentration (mg m^{-3}) in classroom 3	32
Figure S15	$PM_{2.5}$ concentration (mg m^{-3}) in classroom 1	33
Figure S16	$PM_{2.5}$ concentration (mg m^{-3}) in classroom 2	34
Figure S17	$PM_{2.5}$ concentration (mg m^{-3}) in classroom 3	35
Figure S18	VOCs concentration (mg m^{-3}) in classroom 1	36
Figure S19	VOCs concentration (mg m^{-3}) in classroom 2	37
Figure S20	VOCs concentration (mg m^{-3}) in classroom 3	38
Figure S-21	Temperature (ºC) in classroom 1	39
Figure S-22	Temperature (ºC) in classroom 2	40
Figure S-23	Temperature (ºC) in classroom 3	41
Figure S-24	Relative Humidity (%) in classroom 1	42
Figure S-25	Relative Humidity (%) in classroom 2	43
Figure S-26	Relative Humidity (%) in classroom 3	44

Table S1. Sensor specifications.

Gas	Sensor type	Work range (ppm)	Detection limit (ppm)	Resolution (ppm)	Operating conditions	
					Temp (ºC)	RH (%)
CO	GSE[1]	0-100	0.2	0.1	0-40	15-90
CO_2	NDIR[2]	0-5000	10	1	0-40	0-95
HCHO	GSE[1]	0-10	0.01	0.01	0-40	15-90
NO_2	GSE[1]	0-1	0.005	0.001	0-40	15-90
PM	LPC[4]	0.001-1.0 mg/m^3	0.001 mg/m^3	0.001 mg/m^3	0-40	0-90
VOCs	PID[3]	0-20	0.1	0.1	0-40	10-90
O_3	GSE[1]	0-10	0.01	0.01	0-40	15-90

[1] GSE—gas-sensitive electrochemical, [2] NDIR—non-dispersive infra-red, [3] pID—photo ionisation detector, [4] LPC—laser particle counter

Table S2. Field work manual.

**Recruitment, informed consent and questionnaires**
When the students arrived at the arranged date, and before the first lesson, the researchers gave a talk to explain the objectives of the project to them and invited them and the teacher to participate in the study by donating saliva samples. They also remarked that voluntary participation is a legal requirement in this type of study.<br><br>The researchers explained and distributed to the recruited volunteers the following documents: i) an information document for the participant, ii) the informed consent, which was mandatory, had to be signed and included information about requesting consent for the storage of any excess of biological samples in the Biobank for Biomedical Research in Public Health of the Community of Valencia, iii) the procedure and guide for saliva auto-sampling, and iv) questionnaires.<br><br>The questionnaires were divided into three sections as follows: a) the general information of the participants, such as personal information and socio-demographic characteristics; b) viral preventive and protective measures; and c) air quality perception by the donors. The questionnaires were self-filled by the participants during the break time of the sampling days.<br><br>With regards to the protection of the data obtained in this study, this should be in accordance with the current standards on data protection, and participants should be informed of their use. The participants were informed that the information would be published only in scientific journals and would not include any personal details.
**Air and bioaerosol sampling procedure**
To carry out the sampling, the following requirements had to be met:<br><br>- The samples had to be taken under normal conditions of a class day.<br><br>- The equipment had to be placed and connected before the beginning of the classes and turned off (if necessary) at the end of these classes so as not to interfere with the attendees.<br><br>- The sensors had to be placed spaced around the class without interfering with the attendees. They had to be at a similar height to that of the people attending (between 1.5 and 1.7 m) to achieve more realistic results.

- The bioaerosols sampler was placed on the tables among the students. If it was necessary to change the filters during sampling, the said equipment was placed in an easily accessible place so it was as unnoticed as possible during the change.

Saliva sampling procedure

- Before the start of the class, information sheets, questionnaires, sample-taking guides, consent, and the necessary material for taking samples were left on the participant's tables.

- At the beginning of the class's day, there was an explanatory talk about the project to students and teachers, and they were invited to participate voluntarily by donating saliva.

- Information sheets, questionnaires, sample-taking guides, consent to carry out the corresponding analyses, and the storage of samples and the material necessary for taking samples were provided to the participants.

- Whoever wanted to participate completed the questionnaires, signed the consent form, and was able to revoke the storage or use of their sample at any time with a revocation document.

- Sampling was carried out, as explained in Figure 2, during the break or at the end of the classes.

Transport and storage of environmental and biological samples

Air and biological samples correctly labelled were kept at 4ºC in a portable refrigerator, stored zip bags, and in a triple container (UN 3373, Category 3 Biological Substance). Biological samples were transported to the laboratory and analysed within 24 hours of their collection. The excess saliva samples were taken to the Valencian Biobank upon signed informed consent, and they were stored at -80ºC.

Table S3. Results of respiratory viruses in indoor air.

**Virus**	**Results**								
	**Classroom 1**			**Classroom 2**			**Classroom 3**		
	**Day 1**	**Day 2**	**Day 3**	**Day 1**	**Day 2**	**Day 3**	**Day 1**	**Day 2**	**Day 3**
Influenza A	Negative	Negative	Negative	Negative	Negative	Negative	Negative	Negative	Negative
Influenza B	Negative	Negative	Negative	Negative	Negative	Negative	Negative	Negative	Negative
HuCoV 229E	Negative	Negative	Negative	Negative	Negative	Negative	Negative	Negative	Negative
HuCoV NL63	Negative	Negative	Negative	Negative	Negative	Negative	Negative	Negative	Negative
HuCoV OC43	Negative	Negative	Negative	Negative	Negative	Negative	Negative	Negative	Negative
HuCoV HKU1	Negative	Negative	Negative	Negative	Negative	Negative	Negative	Negative	Negative
HMPV-A	Negative	Negative	Negative	Negative	Negative	Negative	Negative	Negative	Negative
HMPV-B	Negative	Negative	Negative	Negative	Negative	Negative	Negative	Negative	Negative
hBoV	Negative	Negative	Negative	Negative	Negative	Negative	Negative	Negative	Negative
Parainfluenza virus 1	Negative	Negative	Negative	Negative	Negative	Negative	Negative	Negative	Negative
Parainfluenza virus 2	Negative	Negative	Negative	Negative	Negative	Negative	Negative	Negative	Negative
Parainfluenza virus 3	Negative	Negative	Negative	Negative	Negative	Negative	Negative	Negative	Negative
Parainfluenza virus 4	Negative	Negative	Negative	Negative	Negative	Negative	Negative	Negative	Negative
AdV	Negative	Negative	Negative	Negative	Negative	Negative	Negative	Negative	Negative
RSV-type A	Negative	Negative	Negative	Negative	Negative	Negative	Negative	Negative	Negative
RSV-type B	Negative	Negative	Negative	Negative	Negative	Negative	Negative	Negative	Negative
HRV/ENV	**Positive**	Negative	Negative	Negative	Negative	Negative	Negative	Negative	Negative

Table S4. List of the tentatively identified substances in indoor air.

**Compound name**	**Molecular formula**	**Toxicological concern**	**Uses, ApplicationsOrigins**
1-(Carboxymethyl)cyclohexanecarboxylic acid	$C_9H_{14}O_4$	Low	Medicine
2H-Pyran-2-methanol, tetrahydro-	$C_6H_{12}O_2$	High	Others
2-Methylbenzoic acid	$C_8H_8O_2$	Low	Industrial
2-Nonanone, 3-(hydroxymethyl)-	$C_{10}H_{20}O_2$	Intermediate	Industrial
2-Pentanone, 4-hydroxy-4-methyl-	$C_6H_{12}O_2$	High	Industrial
2-pentylfuran	$C_9H_{14}O$	High	Cosmetic/Industrial
2-Propanol, 1-(2-methoxypropoxy)-	$C_7H_{16}O_3$	High	Others
3-(4-Isopropylphenyl)-2-methylpropionaldehyde	$C_{13}H_{18}O$	Low	Industrial
3-Acetyl-2,5-dimethyl furan	$C_8H_{10}O_2$	High	Industrial/food
3-Methyladipic acid	$C_7H_{12}O_4$	Low	Biological
4H-Inden-4-one, 1,2,3,5,6,7-hexahydro-1,1,2,3,3-pentamethyl-	$C_{14}H_{22}O$	High	Cosmetics
Amylcinnamaldehyde	$C_{14}H_{18}O$	Low	Cosmetics
Amyl salicylate	$C_{12}H_{16}O_3$	Low	Cosmetics
Benzene, (1-methyldecyl)- [2-phenylundecane]	$C_{17}H_{28}$	Low	Natural product
Benzene, 1,2,3,5-tetramethyl- [isodurene]	$C_{10}H_{14}$	Low	Industrial
Benzene, 1,3-dimethyl- [m-xylene]	C_8H_{10}	Low	Industrial
Benzene, 1-methyl-4-propyl- (toluene)	$C_{10}H_{14}$	Low	Industrial
Benzophenone	$C_{13}H_{10}O$	High	Industrial
Betaine	$C_5H_{11}NO_2$	Low	Medicine/Industrial
Beta-pinene	$C_{10}H_{16}$	Low	Wooden building materials
Bis(2-ethylhexyl) amine	$C_{16}H_{35}N$	High	Industrial
Borneol	$C_{10}H_{18}O$	Low	Medicine
Camphor	$C_{10}H_{16}O$	High	Wooden building materials
CAPSO	$C_9H_{19}NO_4S$	Low	Industrial
Cyclohexene,1-propyl-	C_9H_{16}	Low	Natural product

Cyclopent-2-ene-1-carboxylic acid, 2,3-dimethyl-1-ethyl-, ethyl ester	$C_{12}H_{20}O2$	Low	Pesticide
DEET	$C_{12}H_{17}NO$	Low	Pesticide
Diethyl phthalate	$C_{12}H_{14}O_4$	Low	Cosmetics/Industrial
Ethyl palmitate	$C_{18}H_{36}O_2$	Low	Industrial
Formamide, N,N-dibutyl-	$C_9H_{19}NO$	High	Industrial
Glutaric acid	$C_5H_8O_4$	Low	Industrial
Hexadecanamide	$C_{16}H_{33}NO$	High	Industrial
Indane	C_9H_{10}	High	Industrial
Linalool	$C_{10}H_{18}O$	High	Industrial/insecticide
Limonene	$C_{10}H_{16}$	Low	Cosmetics
Limonene-oxide	$C_{10}H_{16}O$	High	Industrial
Menthol	$C_{10}H_{20}O$	Low	Cosmetics/medicine
Methylsuccinic acid	$C_5H_8O_4$	Low	Industrial
Napthalene	$C_{10}H_8$	High	Industrial
Naphthalene, 1-methoxy-	$C_{11}H_{10}O$	High	Others
Naphthalene, 2-methyl-	$C_{11}H_{10}$	High	Industrial
n-hexyl salicylate	$C_{13}H_{18}O_3$	Low	Industrial
N-Isovalerylglycine	$C_7H_{13}NO_3$	High	Biological
N,N-Dibutyl-formamide	$C_9H_{19}NO$	High	Industrial
NP-014287	$C_{18}H_{32}O_3$	Intermediate	Industrial
NP-018661	$C_8H_{16}O_3$		
NP-018716	$C_{11}H_{20}O_4$	Low	Other
NP-020206	$C_{11}H_{16}O_4$	Low	Industrial
Octan-2-yl-palmitate	$C_{24}H_{48}O_2$	High	Cosmetic/Industrial
p-Cymene	$C_{10}H_{14}$	Low	Industrial
Phenol, 2,6-bis(1,1-dimethylethyl)-4-methyl-, methylcarbamate	$C_{17}H_{27}NO_2$	Intermediate	Cosmetics
Pentanoic acid, 2-hydroxy-4-methyl-, methyl ester	$C_7H_{14}O_3$	Low	Others
Pimelic acid	$C_7H_{12}O_4$	Low	Other

Pregabalin	$C_8H_{17}NO_2$	Low	Medicine/Drug
Propanoic acid, 2-methyl-, 3-hydroxy-2,2,4-trimethylpentyl ester	$C_{12}H_{24}O_3$	Intermediate	Industrial
Pyrimidine-2,4,6-trione, 1-butyl-5-[(2-piperazin-1-yl-ethylamino)methylene]-	$C_{15}H_{25}N_5O_3$	High	Others
Quinazoline-2,4-diol	$C_8H_6N_2O_2$	High	Industrial
Sorbic acid	$C_6H_8O_2$	Low	Industrial
Squalene	$C_{30}H_{50}$	Low	Cosmetics
Suberic acid	$C_8H_{14}O_4$	Low	Industrial
Tributyl acetylcitrate	$C_{20}H_{34}O_8$	Low	Industrial
Triethyl phosphate	$C_6H_{15}O_4P$	High	Industrial
Tropine	$C_8H_{15}NO$	High	Medicine
Urocanic acid	$C_6H_6N_2O_2$	Low	Medicine
Vanillin	$C_8H_8O_3$	Low	Industrial

Table S5. List of the tentatively identified substances in outdoor air.

**Compound name**	**Molecular formula**	**Toxicological concern**	**Uses, applications, or origins**
(-)-Camphanic acid	$C_{10}H_{14}O_4$	High	Industrial
(2R,5R,6R)-3-[(1E,3E)-hepta-1,3-dien-1-yl]-5,6-dihydroxy-2-(hydroxymethyl)cyclohexan-1-one	$C_{14}H_{22}O_4$		
1-(3-Hydroxypropyl)-2-piperidinone	$C_8H_{15}NO_2$	High	Others
1-(tert-butyldimethylsilyl)-3-ethyl-4-isopropyl-1H-pyrrole-2,5-dione	$C_{15}H_{27}NO_2Si$		
1,12-Dodecanediol	$C_{12}H_{26}O_2$	Low	Industrial
1,2,3,4-Tetrahydroisoquinolin-6-ol, 1-[3-hydroxybenzyl]-	$C_{16}H_{17}NO_2$	High	Others
1,2,4-Oxadiazol-5-amine, 3-[4-(cyclopropylamino)-1,2,5-oxadiazol-3-yl]-N-[(4-methoxyphenyl)methyl]-	$C_{15}H_{16}N_6O_3$	High	Others
1,2-Benzenediol, o-(1-adamantanecarbonyl)-	$C_{17}H_{20}O_3$	Low	Others
1,2-Benzenediol, O,O'-di(4-ethylbenzoyl)-	$C_{24}H_{22}O_4$	Low	Others
1,3-Benzenedicarboxylic acid, bis(2-ethylhexyl) ester	$C_{24}H_{38}O_4$	Low	Industrial
1,3-Benzothiazol-2(3H)-one, 3-(3,3-dimethyl-1-oxobutyl)-	$C_{13}H_{15}NO_2S$	High	Others
1,3-Benzoxazole-7-carboxylic acid, 2-methyl-, 4-nitrophenyl ester	$C_{15}H_{10}N_2O_5$	High	Others
1,3-Bis(cinnamoyloxymethyl)adamantane	$C_{30}H_{32}O_4$	High	Others
1,3-Di(2-benzothiazolyl)-1,3-bis(mercaptomethyl)-urea	$C_{17}H_{14}N_4OS_4$	High	Others
1,3-Dimethyl-5-propyl-7-(propene-1-yl)adamantane	$C_{18}H_{30}$	High	Others
1,3-Dioxan-4-one, 2-(1,1-dimethylethyl)-5,6-dimethyl-5-(phenylmethyl)-, [2R-(2α,5α,6α)]-	$C_{17}H_{24}O_3$	High	Others
1,3-Diphenyltriacetin	$C_{21}H_{22}O_6$	High	Pesticide
1,3-Pentanedione, 4,4-dimethyl-1-phenyl-	$C_{13}H_{16}O_2$	Low	Others
1,4,7,-Cycloundecatriene, 1,5,9,9-tetramethyl-, Z,Z,Z-	$C_{15}H_{24}$	Low	Others
1,5-Pentanediol, O,O'-di(2-trifluoromethylbenzoyl)-	$C_{21}H_{18}F_6O_4$	High	Others
1,8,15,22-Tricosatetrayne	$C_{23}H_{32}$	High	Others
1,9-Dioxa-4,12-diazadispiro[4.2.4.2]tetradecane, 3,3,11,11-tetramethyl-	$C_{14}H_{26}N_2O_2$	High	Others
1-[5-(2-Chloro-phenyl)-2-thioxo-[1,3,4]oxadiazol-3-ylmethyl]-piperidine-4-carboxylic acid ethyl ester	$C_{17}H_{20}ClN_3O_3S$	High	Others

13-Docosenamide, (Z)-	$C_{22}H_{43}NO$	High	Industrial
1-(Carboxymethyl)cyclohexanecarboxylic acid	$C_9H_{14}O_4$	Low	Medicine
1-Eicosanol	$C_{20}H_{42}O$	Low	Cosmetics
1H-Cycloprop[e]azulene, 1a,2,3,4,4a,5,6,7b-octahydro-1,1,4,7-tetramethyl-, [1aR-(1aα,4α,4aβ,7bα)]-	$C_{15}H_{24}$	Low	Cosmetics
1-Isopropenyl-2,3-dihydro-1H-benzo[d]imidazol-2-one	$C_{10}H_{10}N_2O$	High	Other
1-Methoxymethyl-1H-benzotriazole	$C_8H_9N_3O$	High	Industrial
1-Methylnicotinamide	$C_7H_8N_2O$	Low	Cosmetic
1-Nonen-3-one, 1-(4-chlorophenyl)-	$C_{15}H_{19}ClO$	High	Others
1-oxo-2,3-dihydro-1H-inden-4-yl benzoate	$C_{16}H_{12}O_3$	High	Other
1-Phenyl-2-butanone	$C_{10}H_{12}O$	Low	Industrial
1-Propanol, 2-(2-methoxypropoxy)-	$C_7H_{16}O_3$	High	Others
2-(2-methoxyethoxy)ethyl 1-phenylcyclopentane-1-carboxylate	$C_{17}H_{24}O_4$		
2-(3,4-Dihydro-5-hydroxy-2-methyl)-2H-1-benzopyranethanol	$C_{13}H_{20}O_2$	High	Others
2(5H)-Furanone, 4-methyl-5,5-bis(2-methyl-2-propenyl)-	$C_{13}H_{18}O_2$	High	Others
2-(Benzylidenehydrazino)-2-oxo-N-(1-phenylethyl)acetamide	$C_{17}H_{17}N_3O_2$	High	Others
2-(Octanoyloxy)propane-1,3-diyl bis(decanoate)	$C_{31}H_{58}O_6$	Low	Others
2,2,3,3,3-Pentafluoro-N-[1-(2-methoxyphenyl)-1-oxopropan-2-yl]-N-methylpropanamide	$C_{14}H_{14}F_5NO_3$	High	Industrial
2,2-dimethyl-N-(4-pyridinyl)propanamide	$C_{10}H_{14}N_2O$	High	Other
2,3-Dihydro-1-benzofuran-2-carboxylic acid	$C_9H_8O_3$	High	Other
2,4-Dinitrophenol	$C_6H_4N_2O_5$	High	Pesticide/Antibacterial drug
2,5-di-tert-Butylhydroquinone	$C_{14}H_{22}O_2$	Low	Industrial
2,5-Pyrrolidinedione, 1-[(4-methylbenzoyl)oxy]-	$C_{12}H_{11}NO_4$	High	Industrial
23-Norcona-5,18(22)-dienin-3-amine, N-methyl-, (3β)-	$C_{22}H_{34}N_2$	High	Others
2-Amino-3-methoxybenzoic acid	$C_8H_9NO_3$	Low	Medicine
2-Cyclohexene-1,4-dione, 5,6-dichloro-2,3-dimethyl-, 1-oxime, o-benzoyl-	$C_{15}H_{13}Cl_2NO_3$	-	Others
2-Ethylhexanoic acid	$C_8H_{16}O_2$	Low	Industrial
2-Furancarboxylic acid, 1-cyclopentylethyl ester	$C_{12}H_{16}O_3$	High	Others
2-Furoylglycine	$C_7H_7NO_4$	High	Industrial

2-Hydroxy-4-(4-hydroxyphenyl)butanoic acid	$C_{10}H_{12}O_4$	Intermediate	Other
2-Hydroxycaproic acid	$C_6H_{12}O_3$	Intermediate	Medicine
2H-1-Benzothiocin-3-methanol, 3,4,5,6-tetrahydro-6-hydroxy-3,5,5-trimethyl-α-[2-(methylthio)phenyl]-	$C_{22}H_{28}O_2S_2$		
2-Methylbenzhydrol, acetate	$C_{16}H_{16}O_2$		
2-Methylbenzoic acid	$C_8H_8O_2$	Low	Industrial
2-methyl-5-nitro-1H-indole	$C_9H_8N_2O_2$	High	Industrial
2-Phenylacetohydrazide	$C_8H_{10}N_2O$	Low	Industrial
2-Propenal, 3-(2,2,6-trimethyl-7-oxabicyclo[4.1.0]hept-1-yl)-	$C_{12}H_{18}O_2$	High	
2-tert-Butylphenol, tert-butyldimethylsilyl ether	$C_{16}H_{28}OSi$		
2-tert-Butyl-4-ethylphenol	$C_{12}H_{18}O$	Low	Other
3-(2-Hydroxyethyl)indole	$C_{10}H_{11}NO$	High	Sedative/Industrial
3-(3,4-Dimethoxyphenyl)lactic acid, ethyl ester, TMS	$C_{16}H_{26}O_5Si$	High	Others
3-(3,7-Dimethyl-octa-2,6-dienyl)-4-hydroxy-benzaldehyde	$C_{17}H_{22}O_2$	Low	Others
3,4-Dimethylbenzoic acid	$C_9H_{10}O_2$	Low	Other
3,4-Dimethoxybenzoylformic acid, TMS	$C_{13}H_{18}O_5Si$	High	Others
3,5-di-tert-Butyl-4-hydroxybenzaldehyde	$C_{15}H_{22}O_2$	Intermediate	Industrial
3-Aminosalicylic acid	$C_7H_7NO_3$	Low	Drug
3-Butylisobenzofuran-1(3H)-one	$C_{12}H_{14}O_2$	High	Medicine
3-Hydroxy-3-(2-oxocyclohexyl)-2-indolinone	$C_{14}H_{15}NO_3$	High	Others
3-Hydroxyphenylacetic acid	$C_8H_8O_3$	Low	Medicine
3-Hydroxyvaleric acid	$C_5H_{10}O_3$	Intermediate	Other
3-Methyl-2-quinoxalinol	$C_9H_8N_2O$	High	Industrial
3-Methylbutyl 4-(dimethylamino)benzoate	$C_{14}H_{21}NO_2$	Low	Cosmetics
3-Methoxyphenylacetic acid	$C_9H_{10}O_3$	Low	Other
3-Phenyl-3-pentanol	$C_{11}H_{16}O$	Low	Industrial
4-(N-Methyl-N-methoxy)indancarboxamide	$C_{12}H_{15}NO_2$	Low	Others
4,8,12,16-tetraoxaeicosan-1-ol	$C_{16}H_{34}O_5$	Low	Others

4-Amino-2,6-dimethyl-3-pyridyl 1-adamantanecarboxylate	$C_{18}H_{24}N_2O_2$	High	Others
4-Ethylbenzoic acid, 2-methoxyethyl ester	$C_{12}H_{16}O_3$	Low	Others
4-Ethylbenzoic acid, 2-methylphenyl ester	$C_{16}H_{16}O_2$	Low	Others
4-Ethylbenzoic acid, cyclobutyl ester	$C_{13}H_{16}O_2$	Low	Others
4-Hydroxy-2-methylpyrrolidine-2-carboxylic acid	$C_6H_{11}NO_3$	High	Industrial
4-Hydroxybenzaldehyde	$C_7H_6O_2$	Low	Industrial
4-Methoxycinnamaldehyde	$C_{10}H_{10}O_2$	Low	Natural
4-Methoxycinnamic acid	$C_{10}H_{10}O_3$	Low	Cosmetic/Industrial
4-Methylumbelliferyl laurate	$C_{22}H_{30}O_4$	High	Others
4-Nitrocatechol	$C_6H_5NO_4$	High	Industrial
4-Nitrophenol	$C_6H_5NO_3$	High	Industrial/Pesticide
4-oxo-5-phenylpentanoic acid	$C_{11}H_{12}O_3$	Low	Natural
4-Phenylbutyric acid	$C_{10}H_{12}O_2$	Low	Medicine/Industrial
4-Phenyl-3-buten-2-one	$C_{10}H_{10}O$	Low	Industrial
4-tert-Butylbenzenethiol, S-acetyl-	$C_{12}H_{16}OS$	Low	Industrial
5-(2,2-Dimethyl-[1,3]dioxolan-4-yl)-4-(2-hydroxyethyl)-1,2-dimethyl-pyrazolidin-3-one	$C_{12}H_{22}N_2O_4$	High	Others
5,10-Pentadecadien-1-ol, (Z,Z)-	$C_{15}H_{28}O$	Low	Others
5,7a-Didehydroindicine pertrimethylsilyl ether	$C_{24}H_{47}NO_5Si_3$	High	Others
5-Hydroxytryptophan	$C_{11}H_{12}N_2O_3$	High	Medicine
6-(4-phenylpiperazino)hexanoic acid hydrochloride	$C_{16}H_{24}N_2O_2$	High	Other
6-Methoxyquinoline	$C_{10}H_9NO$	High	Industrial
6-Methylquinoline	$C_{10}H_9N$	High	Industrial
7-Oxabicyclo[4.1.0]heptane, 1-methyl-4-(2-methyloxiranyl)-	$C_{10}H_{16}O_2$	High	Industrial
8,11-Dimethyl-2,9,10-trioxa-6-azonia-1-boratatricyclo[4.3.3.0(1,6)]dodecane	$C_9H_{18}BNO_3$		
8-Heptadecanol, 8-methyl-	$C_{18}H_{38}O$	High	Others
Acetamide, 2-phenyl-N-[1-(4-hydroxybenzyl)-1-cyclohexyl)-	$C_{21}H_{25}NO_2$	High	Others
Acetic acid 2-oxo-2,3-dihydro-1H-benzo[e][1,4]diazepin-3-yl ester	$C_{11}H_{10}N_2O_3$	High	Others
Adamantane-1-carboxamide, N-(4-methyl-3-furazanyl)-	$C_{14}H_{19}N_3O_2$	High	Others

Adipic acid, di(2-fluorophenyl) ester	$C_{18}H_{16}F_2O_4$	High	Industrial
Alanine, N-methyl-N-(2-methoxyethoxycarbonyl)-, isobutyl ester	$C_{12}H_{23}NO_5$	High	Others
Alloaromadendrene	$C_{15}H_{24}$	Low	Industrial
Alverine	$C_{20}H_{27}N$	High	Medicine
Arctiol	$C_{15}H_{26}O_2$	High	Others
Benzamide, 4-chloro-3-methyl-N-allyl-N-ethyl-	$C_{13}H_{16}ClNO$		
Benzamide, N-(2-chloro-3-pyridyl)-4-ethoxy-	$C_{14}H_{13}ClN_2O_2$	High	Others
Benzamide	C_7H_7NO	Low	Industrial
Benzene, (1,1,4,6,6-pentamethylheptyl)-	$C_{18}H_{30}$	Low	Others
Benzene, (1,1-dimethylnonyl)-	$C_{17}H_{28}$	Low	Others
Benzene, 1-ethyl-2,3-dimethyl-	$C_{10}H_{14}$	Low	Others
benzenemethanamine, N,N-bis(4-methylphenyl)-	$C_{21}H_{21}N$	High	Others
Benzoic acid, 4-(4-butylcyclohexyl)-, 2,3-dicyano-4-ethoxyphenyl ester	$C_{27}H_{30}N_2O_3$	High	Others
Benzoic acid, 4-amino-, 4-acetoxy-2,2,6,6-tetramethyl-1-piperidinyl ester	$C_{18}H_{26}N_2O_4$	High	Others
Benzoic acid, 4-amino-, 4-hydroximino-2,2,6,6-tetramethyl-1-piperidinyl ester	$C_{16}H_{23}N_3O_3$	High	Others
Benzoic acid, octyl ester	$C_{15}H_{22}O_2$	Low	Cosmetics
Benzoic acid	$C_7H_6O_2$	Low	Industrial
Benzothiazole	C_7H_5NS	High	Industrial
Bioresmethrin	$C_{22}H_{26}O_3$	High	Pesticide
BMK glycidic acid	$C_{10}H_{10}O_3$	High	Industrial
Butyl citrate	$C_{18}H_{32}O_7$	High	Industrial
Carbaryl	$C_{12}H_{11}NO_2$	High	Pesticide
Catechol	$C_6H_6O_2$	Low	Industrial
cis-11-Eicosenamide	$C_{20}H_{39}NO$	High	Others
cis-8-Isopropylbicyclo[4.3.0]non-3-ene	$C_{12}H_{20}$	High	Others
cis-Carveol, O-(pentafluoropropionyl)-	$C_{13}H_{15}F_5O_2$		
Citroflex 4	$C_{18}H_{32}O_7$	High	Industrial
Cyclohexane, 1-(cyclohexylmethyl)-2-ethyl-, cis-	$C_{15}H_{28}$	Intermediate	Others

Cyclohexane, 1,1'-methylenebis-	$C_{13}H_{24}$	Intermediate	Industrial
Cyclohexanecarboxylic acid, 4-butyl-, 4-methoxyphenyl ester	$C_{18}H_{26}O_3$	Low	Others
Cycloheximide	$C_{15}H_{23}NO_4$	High	Pesticide
Cyclopropanecarboxylic acid, 1-(phenylmethyl)-, 2,6-bis(1,1-dimethylethyl)-4-methylphenyl ester	$C_{26}H_{34}O_2$	High	Others
Cyclopropanecarboxylic acid, 2-methyl-1-(phenylmethyl)-, 2,6-bis(1,1-dimethylethyl)-4-methylphenyl ester	$C_{27}H_{36}O_2$	High	Others
Decanedioic acid, dibutyl ester	$C_{18}H_{34}O_4$	Low	Industrial
Dibenzoylmethane	$C_{15}H_{12}O_2$	High	Industrial
Didemethylisoproturon	$C_{10}H_{14}N_2O$	Low	Pesticide
Di-n-octyl phthalate	$C_{24}H_{38}O_4$	Low	Industrial
dl-7-Azatryptophan	$C_{10}H_{11}N_3O_2$	High	Industrial
DL-Alanine, N-methyl-N-(2-ethylhexyloxycarbonyl)-, heptadecyl ester	$C_{30}H_{59}NO_4$	Low	Others
D-Panthenol	$C_9H_{19}NO_4$	High	Medicine
Eicosanoic acid, 2-(acetyloxy)-1-[(acetyloxy)methyl]ethyl ester	$C_{27}H_{50}O_6$	Low	Others
Ethaneperoxoic acid, 1-cyano-1-[2-(2-phenyl-1,3-dioxolan-2-yl)ethyl]pentyl ester	$C_{19}H_{25}NO_5$	High	Others
Formetorex	$C_{10}H_{13}NO$	Low	Medicine/Industrial
Fumaric acid, octyl pent-4-en-2-yl ester	$C_17H_{28}O_4$	Low	Others
Furazan-3-carboxylic acid, 4-amino-, 2-(2,5-dimethylphenyl)-2-oxoethyl ester	$C_{13}H_{13}N_3O_4$	High	Others
Gabapentin	$C_9H_{17}NO_2$	High	Medicine
Glutaric anhydride	$C_5H_6O_3$	High	Industrial
Glycine, N-(3-methyl-1-oxo-2-butenyl)-, methyl ester	$C_8H_{13}NO_3$	High	Others
Glycylglycine, N,N'-diethyl-N'-(2-methoxyethoxycarbonyl)-, propyl ester	$C_{15}H_{28}N_2O_6$	High	Others
Heptanamide, N-phenyl-	$C_{13}H_{19}NO$	Low	Industrial
Hexa(methoxymethyl)melamine	$C_{15}H_{30}N_6O_6$	High	Industrial
Hexacosane	$C_{26}H_{54}$	Low	Industrial
Hexadecanoic acid, 2-hydroxyethyl ester	$C_{18}H_{36}O_3$	Low	Cosmetics
Hexanamide, 3,5,5-trimethyl-N-propyl-N-heptyl-	$C_{19}H_{39}NO$		Others
Hexanamide, N-(2-phenylethyl)-N-tetradecyl-	$C_{28}H_{49}NO$		

Hinesol	$C_{15}H_{26}O$	High	Medicine
Homoserine lactone 1-(dimethylamino)naphthalene-5-sulfonamide	$C_{16}H_{18}N_2O_4S$	High	Others
Hydrocinnamic acid	$C_9H_{10}O_2$	Low	Cosmetic
Isobutyl dehydroabietate	$C_{24}H_{36}O_2$		
L-(-)-Arabitol	$C_5H_{12}O_5$	Low	Industrial
L-Cysteinesulfinic acid	$C_3H_7NO_4S$	High	Biological
L-Iditol	$C_6H_{14}O_6$	Low	Industrial
Levulinic acid	$C_5H_8O_3$	Low	Industrial
Linoelaidic acid	$C_{18}H_{32}O_2$	Low	Medicine
L-Leucine, N-(3-methylbutyl)-, methyl ester	$C_{12}H_{25}NO_2$	High	
Metalaxyl	$C_{15}H_{21}NO_4$	Low	Pesticide/Antibacterial drug
Metamfepramone	$C_{11}H_{15}NO$	Low	Medicine/drug
Methyl salicylate	$C_8H_8O_3$	Low	Medicine
Monomethyl phthalate	$C_9H_8O_4$	Low	Industrial
N-(2,4-Dimethylphenyl)formamide	$C_9H_{11}NO$	Low	Amitraz metabolite
N'-(1,5-Dimethylpyrazol-4-ylsulfonyl)quinoline-2-carbohydrazide	$C_{15}H_{15}N_5O_3S$		
N-(1-Benzyl-2-methylpyrrolidin-3-yl)-5-chloro-2-methoxy-4-(methylamino)benzamide	$C_{21}H_{26}ClN_3O_2$	High	Medicine
N'-(2,2-Dimethylpropanoyl)-2,2-dimethyl-N-(naphthalen-1-yl)propanehydrazide	$C_{20}H_{26}N_2O_2$		
N,N-Diethylethanolamine	$C_6H_{15}NO$	Low	Industrial
N,N'-Bis(2,6-dimethyl-6-nitrosohept-2-en-4-one)	$C_{18}H_{30}N_2O_4$	High	Others
Naphthalene, 1,2,3,4-tetrahydro-5,8-dimethyl-1-octyl-	$C_{20}H_{32}$	Low	Others
N-Behenoyl-5-hydroxytryptamine	$C_{32}H_{54}N_2O_2$	High	Others
N-Benzylformamide	C_8H_9NO	Low	Industrial
N-Methyl-N-methoxy-5,6,7,8-tetrahydro-1-naphtamide	$C_{13}H_{17}NO_2$	Low	Others
N-Methylcaprolactam	$C_7H_{13}NO$	High	Industrial
Nicotinuric acid	$C_8H_8N_2O_3$	High	Other
Normeperidine	$C_{14}H_{19}NO_2$	High	Other
NP-002089	$C_{15}H_{22}O_3$	High	Other

NP-012551	$C_8H_8O_4$		
NP-012972	$C_{14}H_{24}O_3$	Intermediate	Industrial
NP-022068	$C_8H_{12}O_5$	High	Other
Octadecanoic acid, 2-hydroxyethyl ester	$C_{20}H_{40}O_3$	Low	Industrial
Oleyl alcohol, methyl ether	$C_{19}H_{38}O$	Low	Others
Oxiranecarboxylic acid, 3-methyl-3-phenyl-, ethyl ester, cis-	$C_{12}H_{14}O_3$	High	Others
Panthenol	$C_9H_{19}NO_4$	High	Medicine
PEG Monolaurate n5	$C_{22}H_{44}O_7$	High	Other
PEG n5	$C_{10}H_{22}O_6$	High	Industrial
PEG n6	$C_{12}H_{26}O_7$	High	Industrial
PEG n7	$C_{14}H_{30}O_8$	High	Industrial
PEG n8	$C_{16}H_{34}O_9$	High	Industrial
Perillic acid	$C_{10}H_{14}O_2$	Low	Medicine
Phenmetrazine	$C_{11}H_{15}NO$	High	Medicine/drug/industrial
Phenylglyoxylic acid	$C_8H_6O_3$	Low	Industrial
Phenylpyruvic acid	$C_9H_8O_3$	Low	Industrial
Phosphinic amide, N,N-dimethyl-, bis(2-phenylhydrazino)-	$C_{14}H_{20}N_5OP$	High	Others
Phthaldialdehyde	$C_8H_6O_2$	Low	Industrial
Phthalic acid, di-(1-hexen-5-yl) ester	$C_{20}H_{26}O_4$	Low	Others
Phthalic acid, isohexyl 8-quinolinyl ester	$C_{23}H_{23}NO_4$	High	Others
Phthalimide, N-(2-hydroxy-3-butyl)-	$C_{12}H_{13}NO_3$	High	Others
Phthalimidoacetic acid	$C_{10}H_7NO_4$	High	Others
PPG n5	$C_{15}H_{32}O_6$	High	Industrial
Propane, 2,2'-[methylenebis(oxy)]bis[2-methyl-	$C_9H_{20}O_2$	Low	Others
Propanoic acid, 2-(benzoylamino)-3-(benzoyloxy)-, 1-methylethyl ester	$C_{20}H_{21}NO_5$	Low	Others
Propionamide, 3-cyclopentyl-N-(2-butyl)-N-nonyl-	$C_{21}H_{41}NO$		
Pyridine, 2-(bis[bis(diethylamino)phosphino]methyl)-6-methyl-	$C_{23}H_{47}N_5P_2$	High	Others
Pyrimethanil	$C_{12}H_{13}N_3$	High	Pesticide

Pyrimidine, 4,6-dimethoxy-5-acetyl-	$C_8H_{10}N_2O_3$	High	Others
Ricinine	$C_8H_8N_2O_2$	High	Pesticide/Industrial
Salicylic acid	$C_7H_6O_3$	Low	Medicine
Sulfurous acid, hexyl nonyl ester	$C_{15}H_{32}O_3S$	High	Others
Terephthalic acid, di(1-cyclopentylethyl) ester	$C_{22}H_{30}O_4$	Low	Others
Tetradecanamide	$C_{14}H_{29}NO$	High	Industrial
Tetradecanoic acid, 2-hydroxyethyl ester	$C_{16}H_{32}O_3$	Low	Others
Tetranor-12(S)-HETE	$C_{16}H_{26}O_3$	Low	Medicine
trans-11-Tetradecenyl acetate	$C_{16}H_{30}O_2$	Low	Pesticide
trans-3-Azido-1,2,3,4-tetrahydro-2-naphthyl methanesulfonate	$C_{11}H_{13}N_3O_3S$	Low	Others
trans-Cinnamic acid	$C_9H_8O_2$	Low	Industrial
Tributyl acetylcitrate	$C_{20}H_{34}O_8$	Low	Medicine
Triethanolamine	$C_6H_{15}NO_3$	Low	Industrial/Cosmetics
Trimethyl-(2-phenoxymethoxyethyl)silane	$C_{12}H_{20}O_2Si$	High	Others
Triisopropanolamine	$C_9H_{21}NO_3$	Low	Industrial
Tyrosol	$C_8H_{10}O_2$	Low	Other
Vanillin	$C_8H_8O_3$	Low	Industrial
Valpromide	$C_8H_{17}NO$	High	Medicine
Vinyl caprylate	$C_{10}H_{18}O_2$	Low	Industrial
Vinyldimethyl(acetoxymethyl)silane	$C_7H_{14}O_2Si$	High	Others

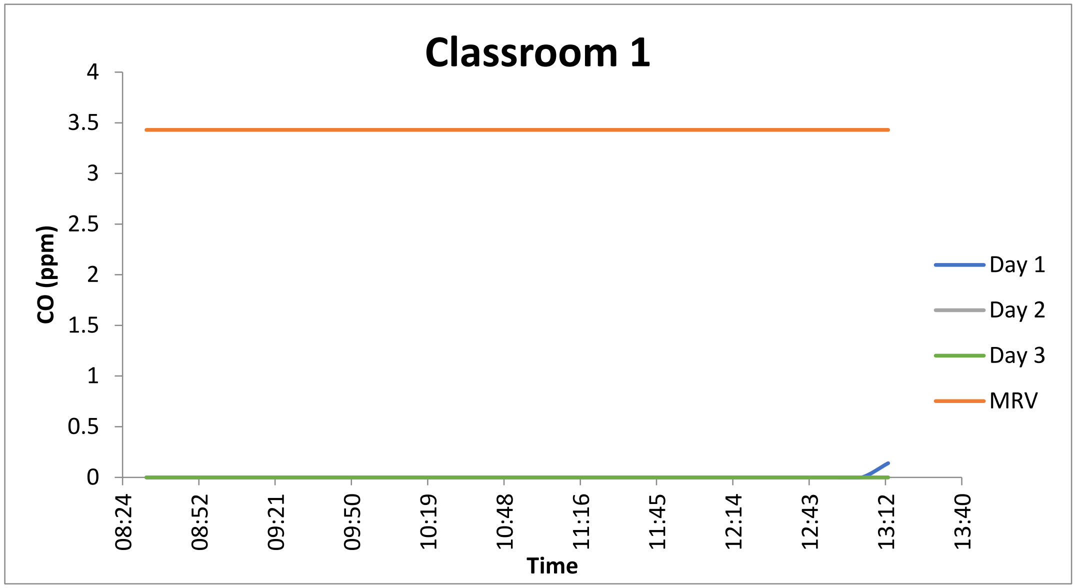

Figure S1. CO concentration (ppm) in classroom 1 (MRV = maximum recommended value).

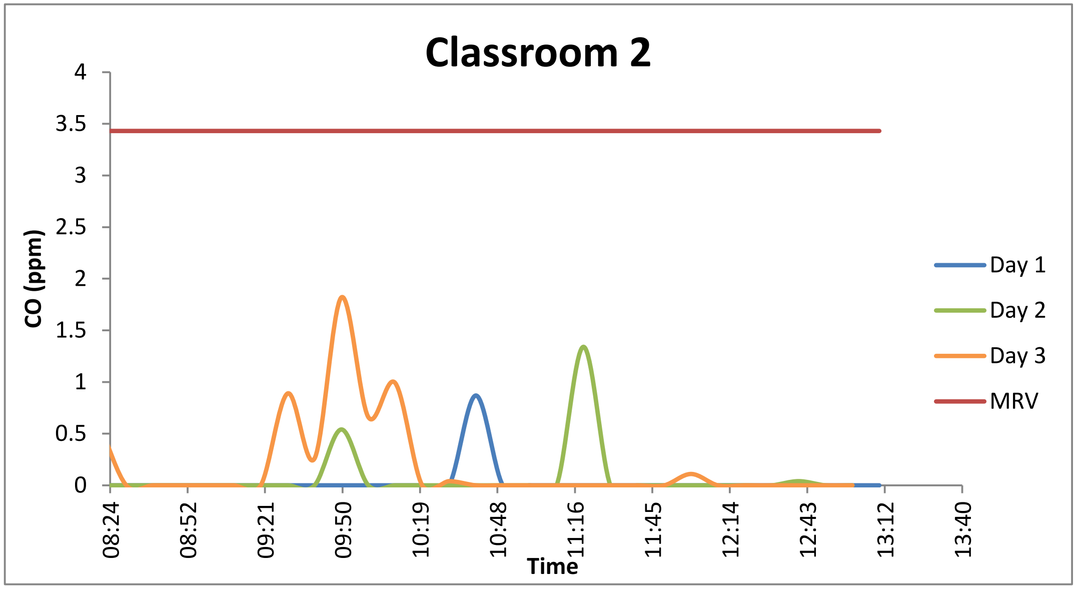

Figure S2. CO concentration (ppm) in classroom 2 (MRV = maximum recommended value).

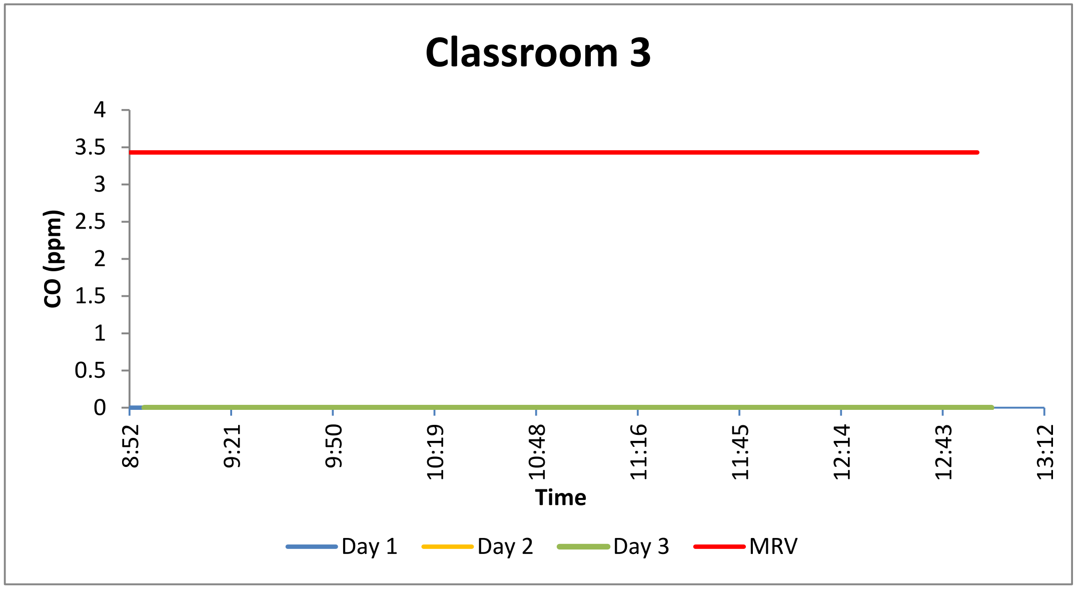

Figure S3. CO concentration (ppm) in classroom 3 (MRV = maximum recommended value).

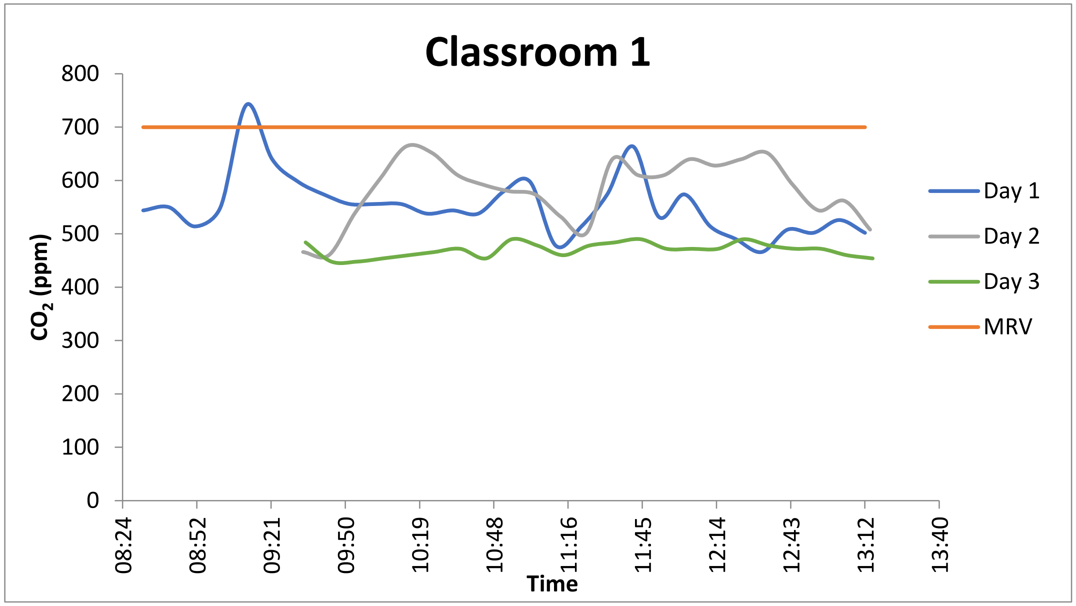

Figure S4. CO_2 concentration (ppm) in classroom 1 (MRV = maximum recommended value).

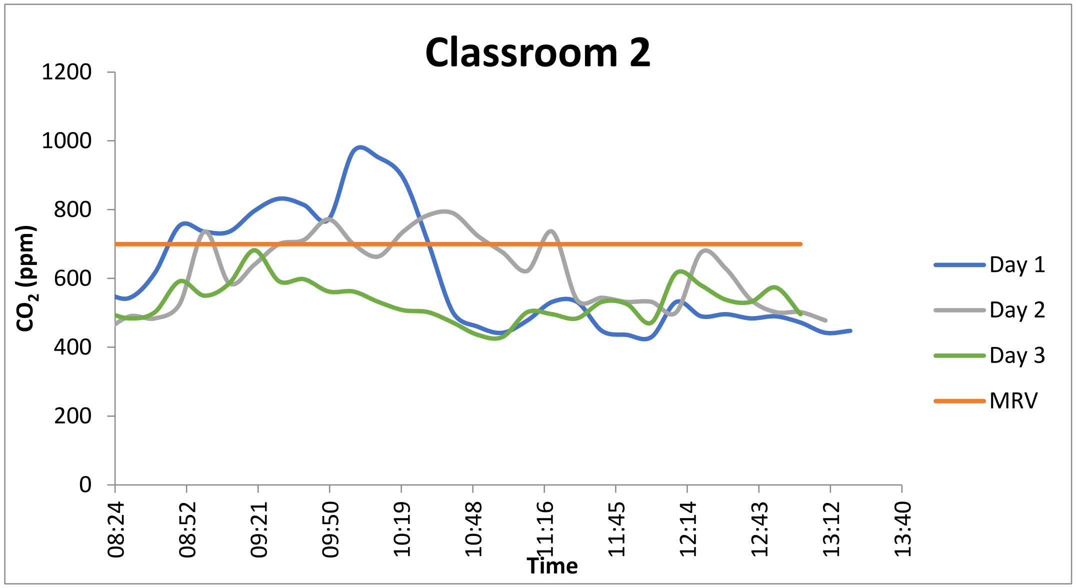

Figure S5. CO_2 concentration (ppm) in classroom 2 (MRV = maximum recommended value).

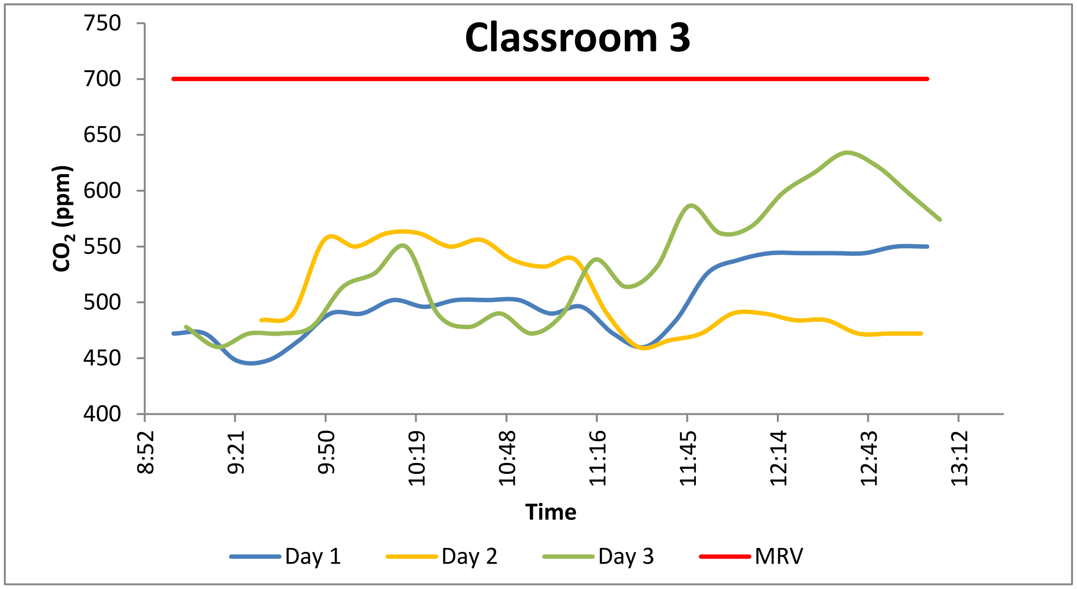

Figure S6. CO_2 concentration (ppm) in classroom 3 (MRV = maximum recommended value).

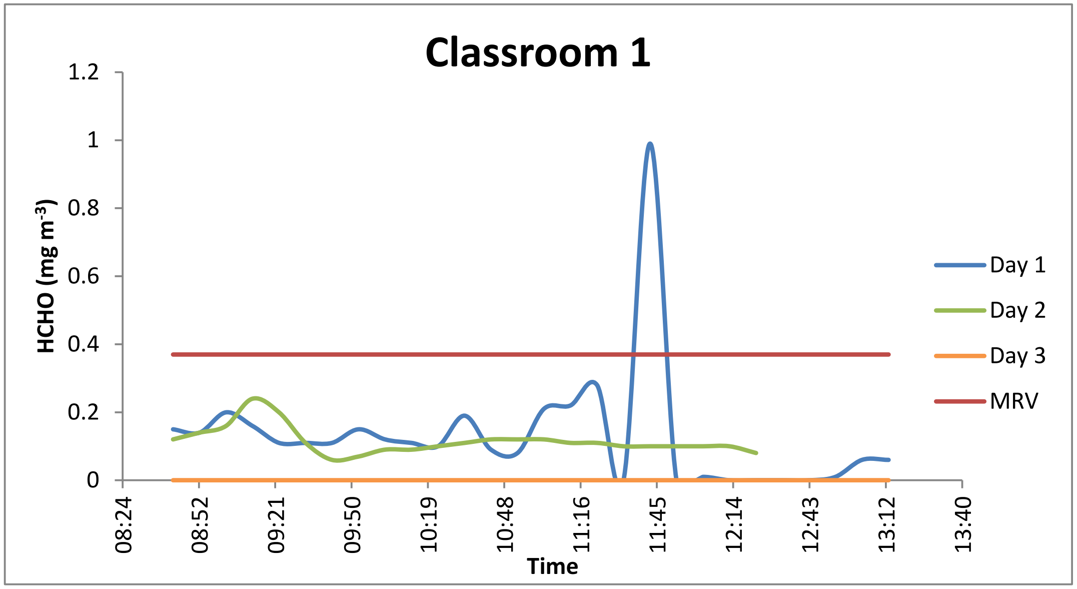

Figure S7. HCHO concentration (mg m^{-3}) in classroom 1 (MRV = maximum recommended value).

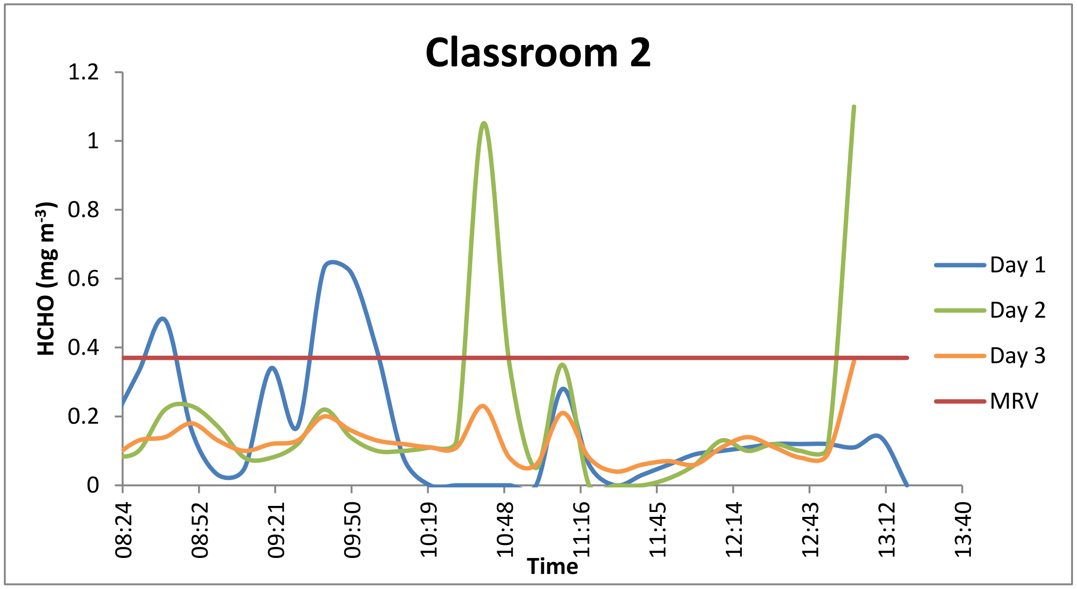

Figure S8. HCHO concentration (mg m^{-3}) in classroom 2 (MRV = maximum recommended value).

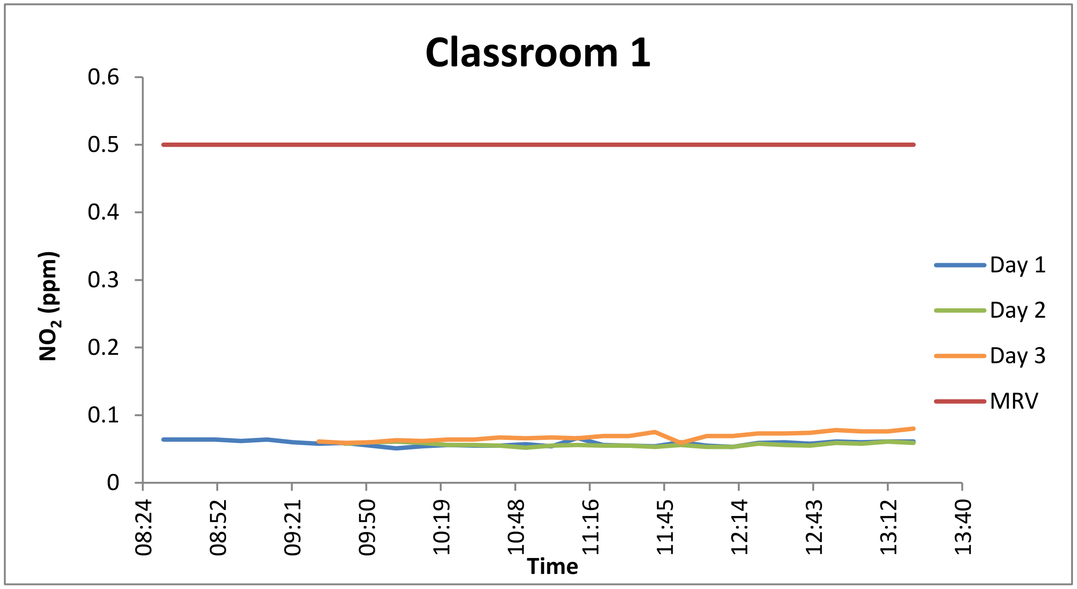

Figure S9. NO_2 concentration (ppm) in classroom 1 (MRV = maximum recommended value).

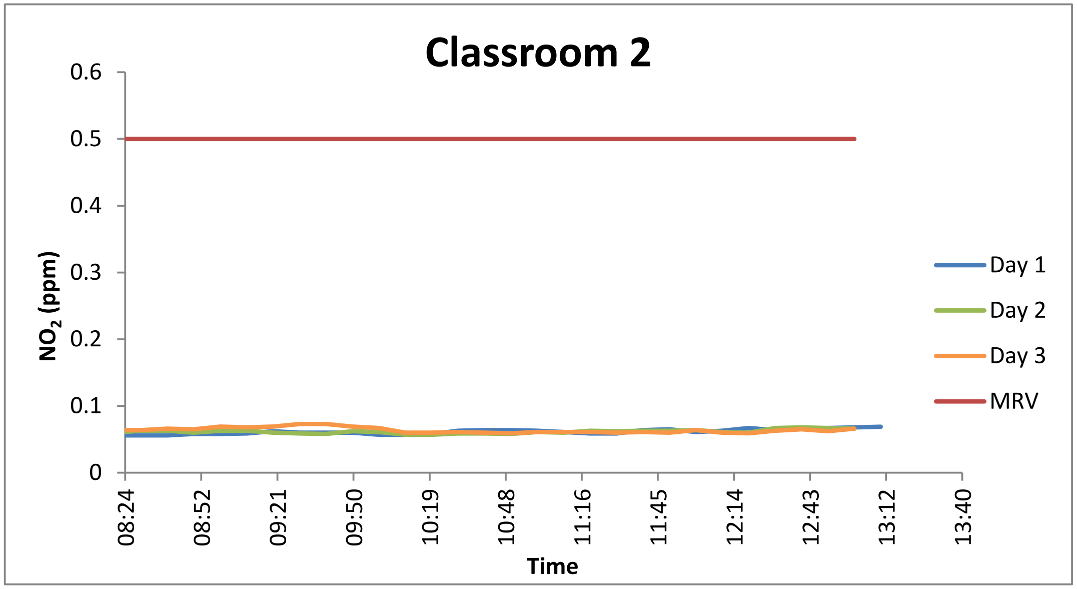

Figure S10. NO_2 concentration (ppm) in classroom 2 (MRV = maximum recommended value).

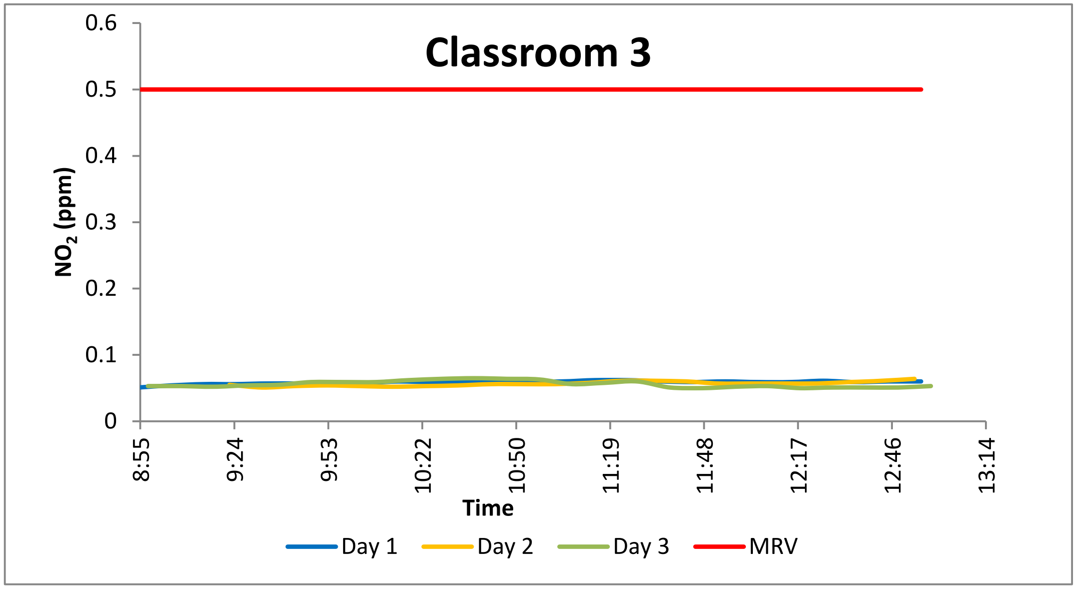

Figure S11. NO_2 concentration (ppm) in classroom 3 (MRV = maximum recommended value).

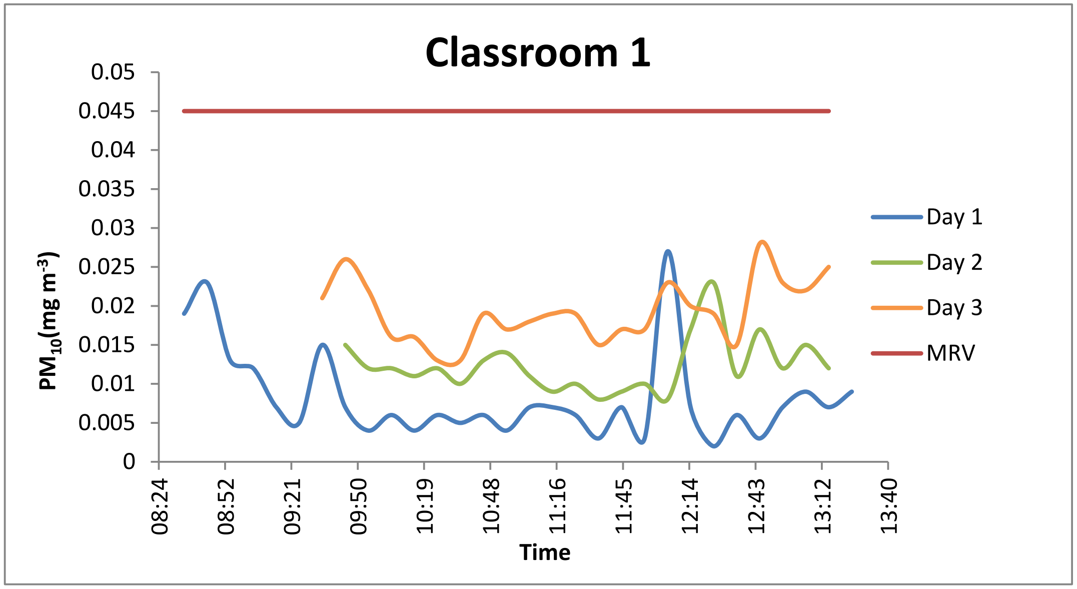

Figure S12. PM_{10} concentration (mg m^{-3}) in classroom 1 (MRV = maximum recommended value).

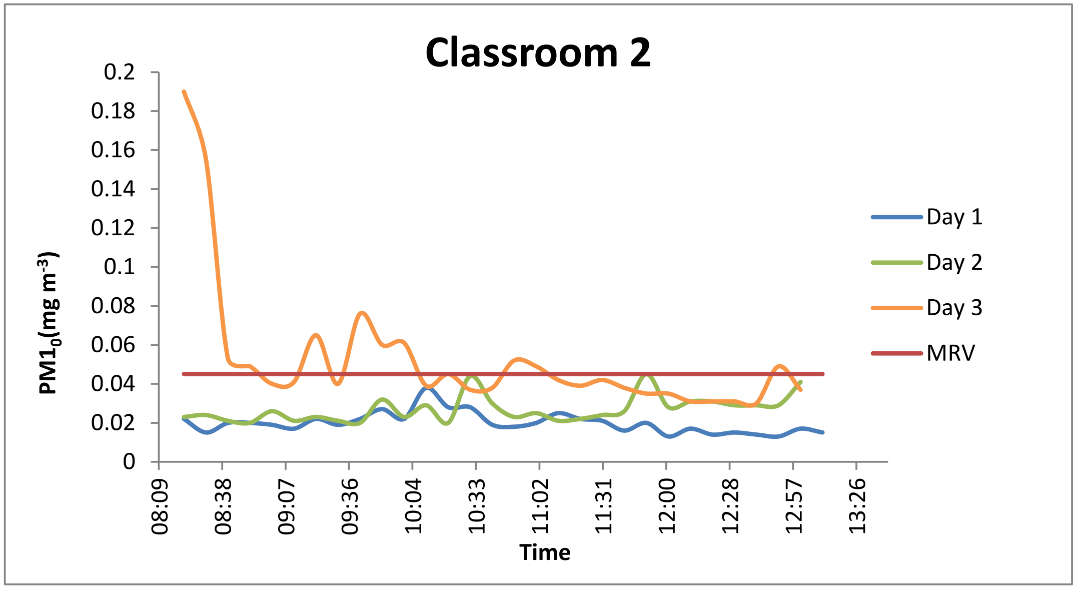

Figure S13. PM_{10} concentration (mg m^{-3}) in classroom 2 (MRV = maximum recommended value).

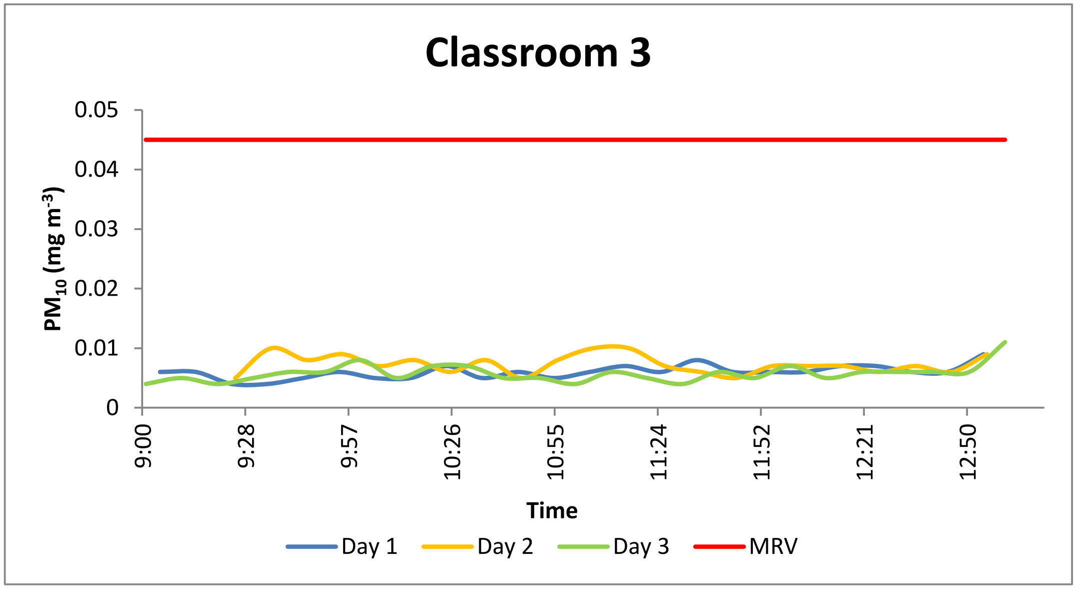

Figure S14. PM_{10} concentration (mg m^{-3}) in classroom 3(MRV = maximum recommended value).

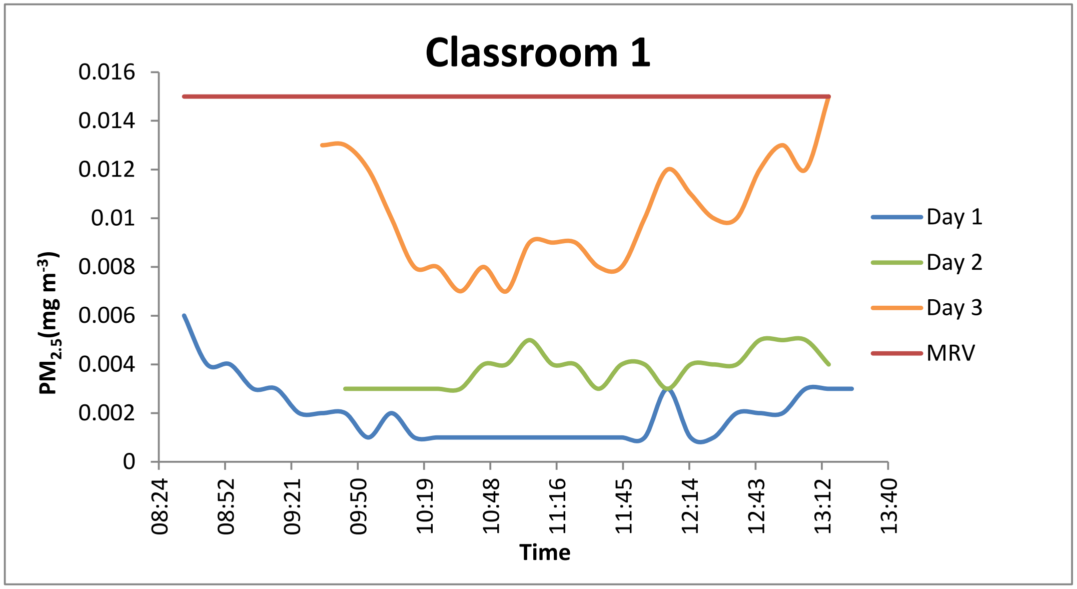

Figure S15. $PM_{2.5}$ concentration (mg m^{-3}) in classroom 1 (MRV = maximum recommended value).

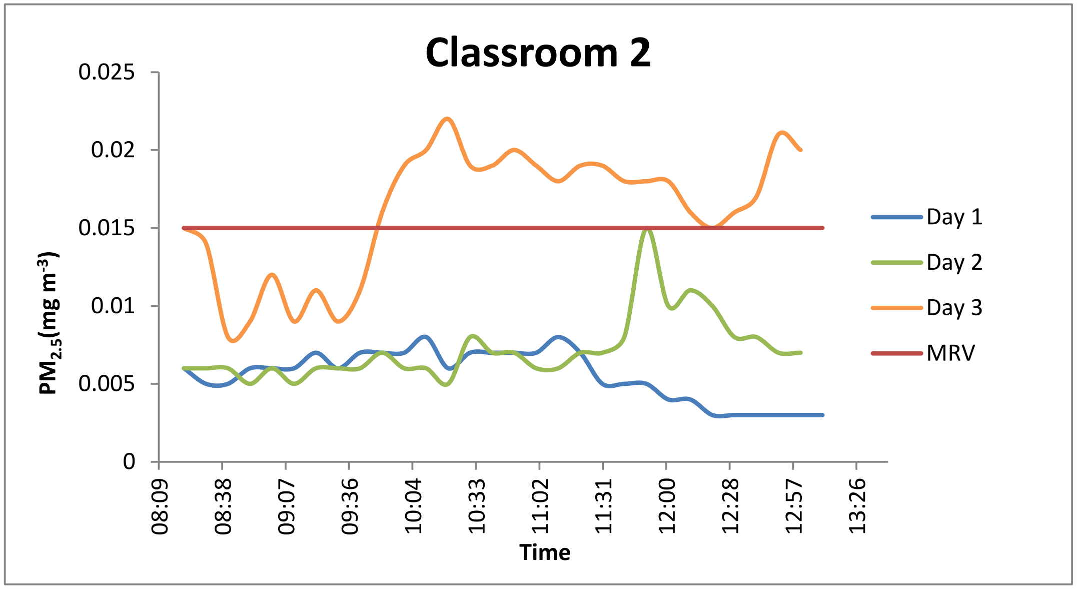

Figure S16. $PM_{2.5}$ concentration (mg m^{-3}) in classroom 2 (MRV = maximum recommended value).

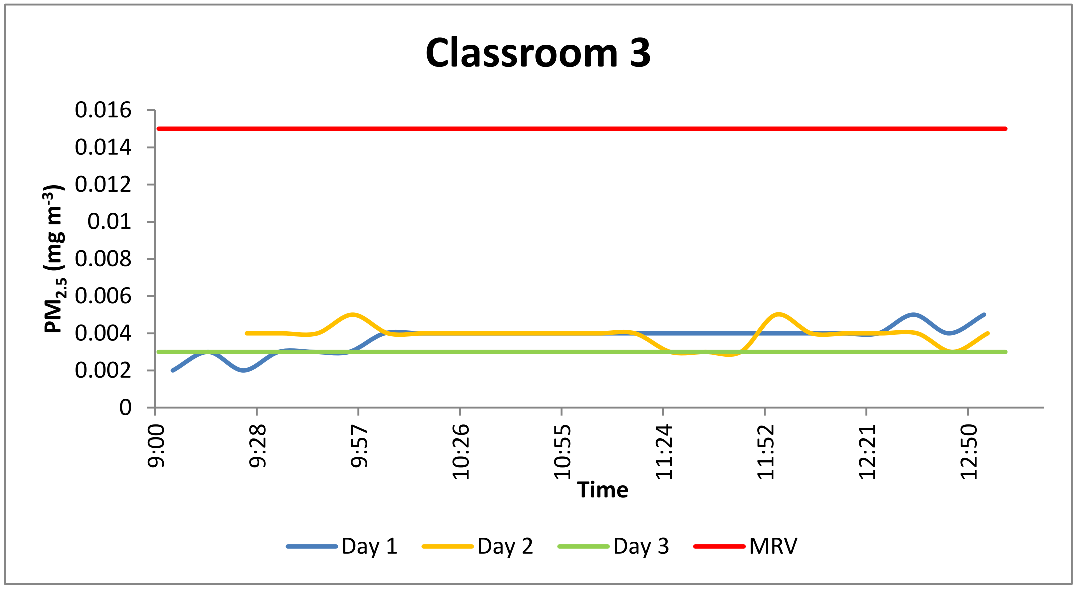

Figure S17. $PM_{2.5}$ concentration (mg m^{-3}) in classroom 3 (MRV = maximum recommended value).

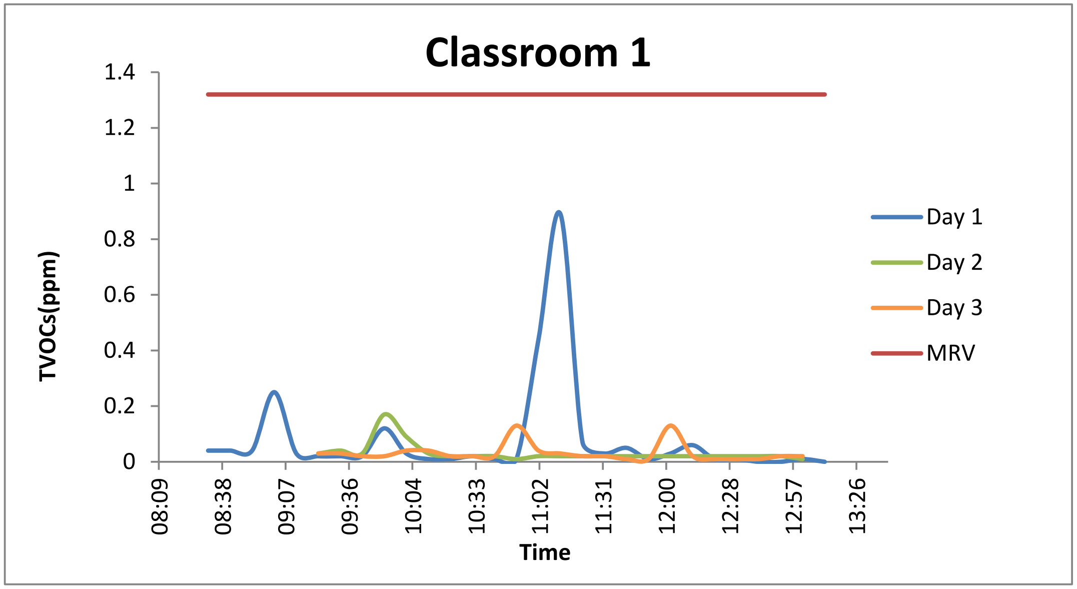

Figure S18. VOC concentrations (ppm) in classroom 1 (MRV = maximum recommended value).

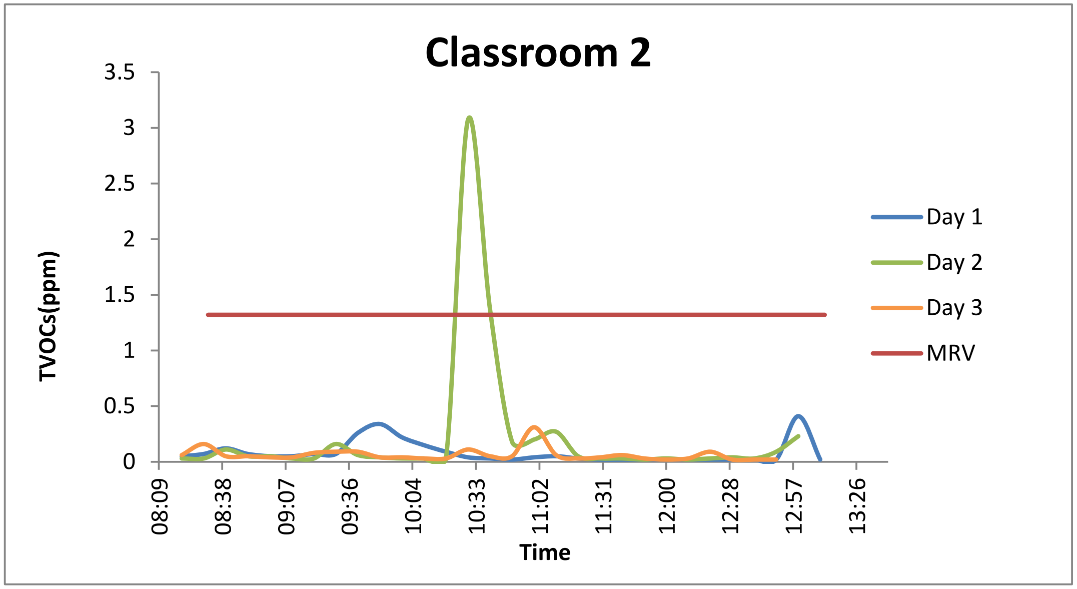

Figure S19. VOC concentrations (ppm) in classroom 2 (MRV = maximum recommended value).

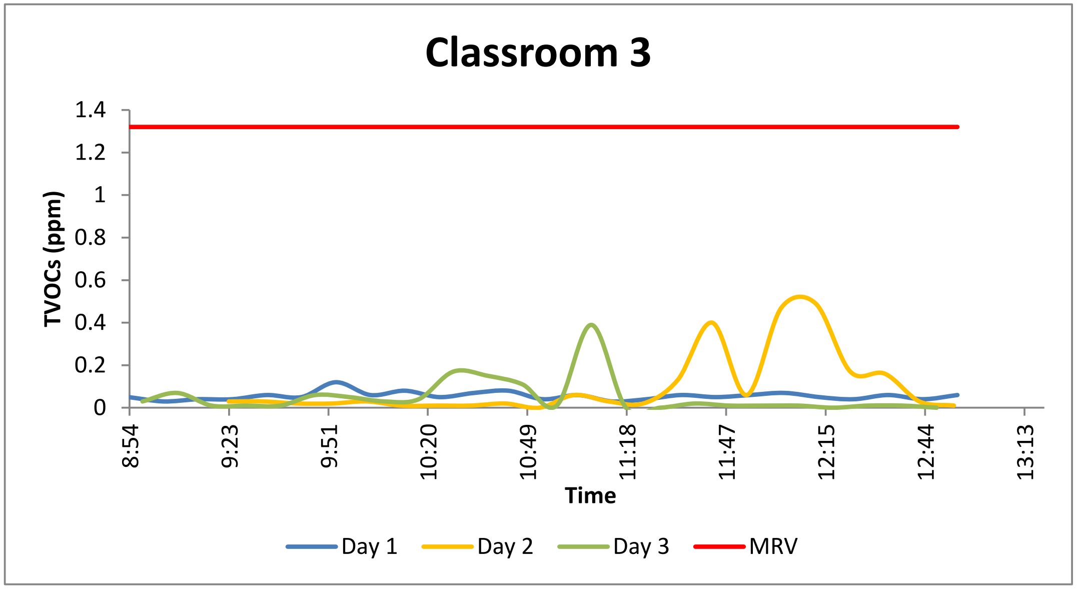

Figure S20. VOC concentrations (ppm) in classroom 3 (MRV = maximum recommended value).

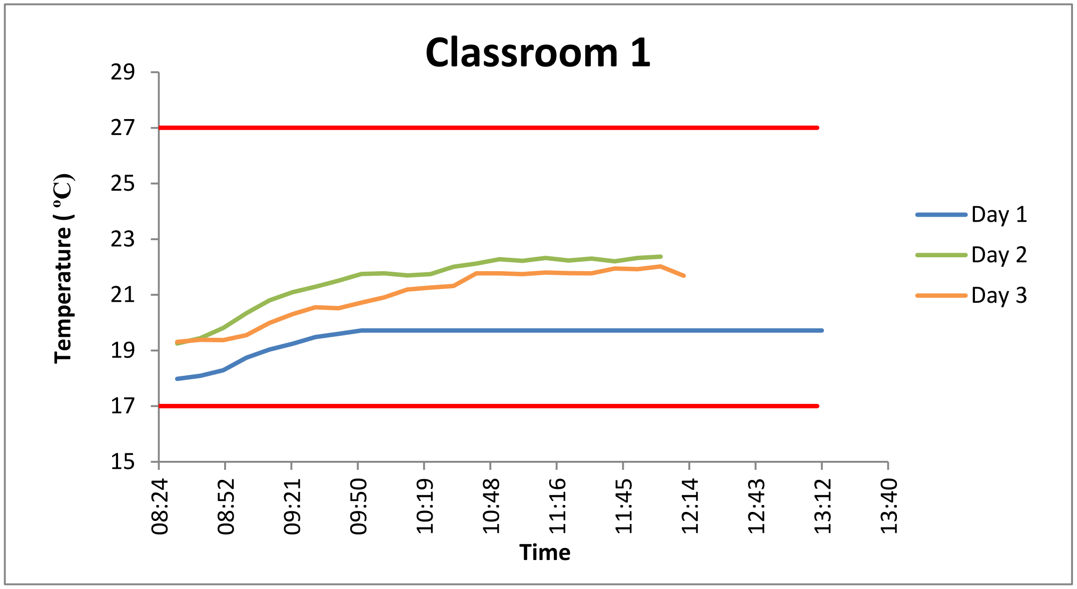

Figure S21. Temperature (ºC) in classroom 1.

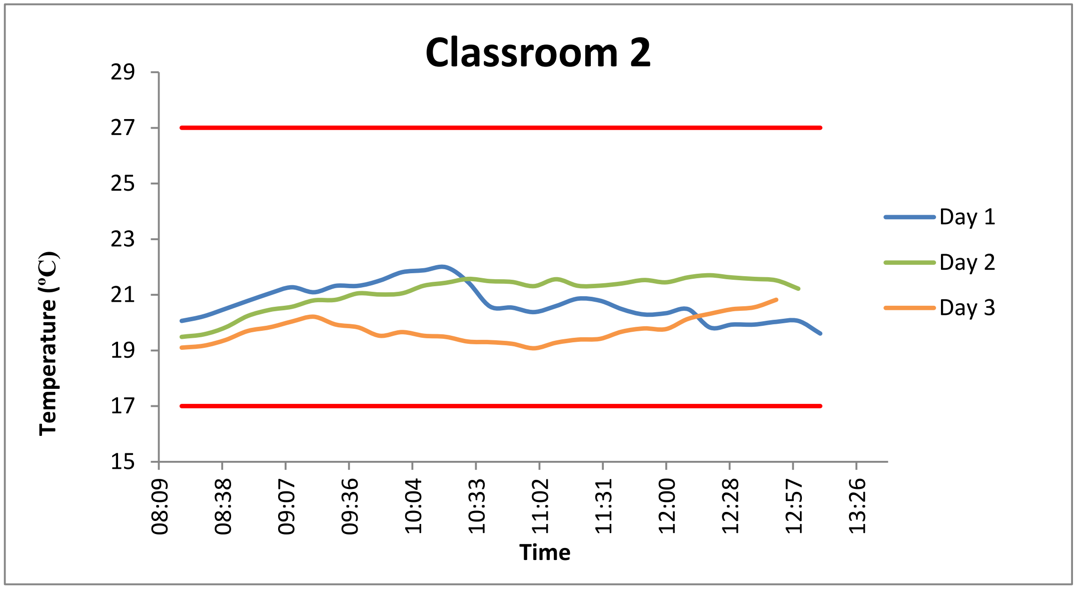

Figure S22. Temperature (ºC) in classroom 2.

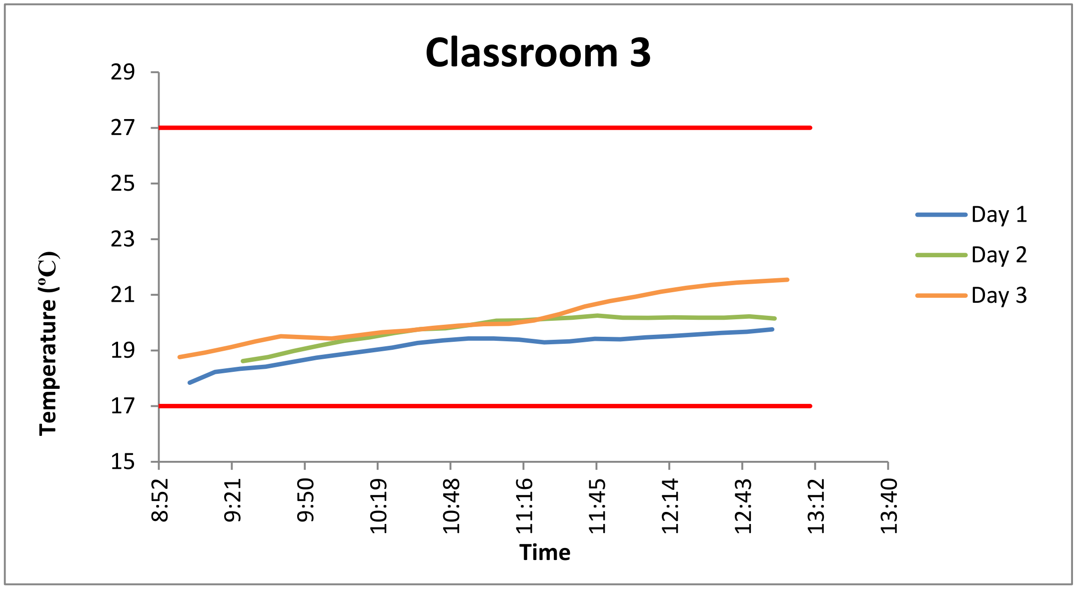

Figure S23. Temperature (ºC) in classroom 3.

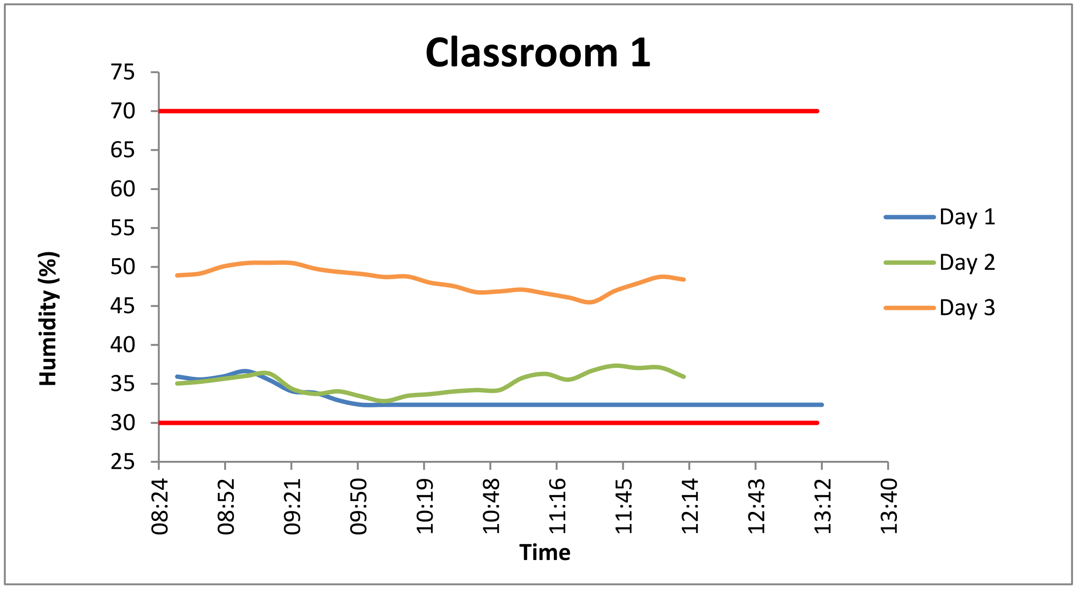

Figure S24. Relative Humidity (%) in classroom 1.

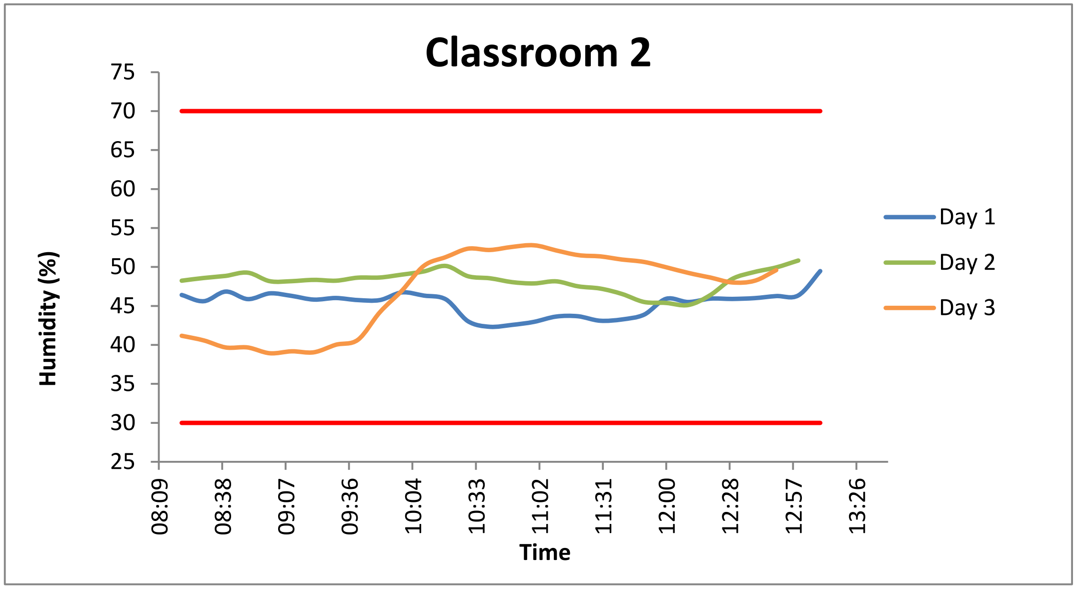

Figure S25. Relative humidity (%) in classroom 2.

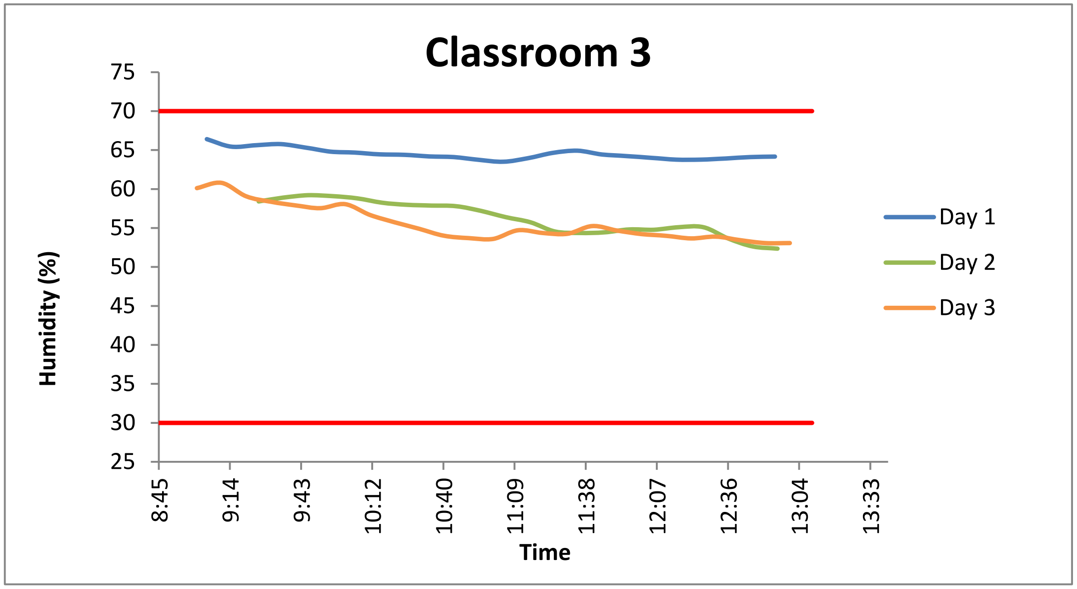

Figure S26. Relative humidity (%) in classroom 3.
